# Comparative efficacy of different mind–body exercises on functional capacity and quality of life in patients with chronic heart failure: a systematic review and network meta-analysis

**DOI:** 10.3389/fpubh.2026.1802127

**Published:** 2026-05-01

**Authors:** Yongliang Zhu, Haozhe Wang, Jiayi Yao, Shiguan Jia, Bin Gai, Junhao He, Peng Suo

**Affiliations:** 1 School of Physical Education, Shandong Sport University, Jinan, China; 2 School of Physical Education, China University of Mining and Technology, Xuzhou, China; 3 School of Physical Education and Training, Capital University of Physical Education and Sports, Beijing, China

**Keywords:** chronic heart failure, exercise tolerance, mind–body exercise, network meta-analysis, quality of life, randomized controlled trials

## Abstract

**Background and objective:**

Mind–body exercise (MBE) has emerged as a vital adjunctive modality in the rehabilitation of chronic heart failure (CHF). However, the relative advantages of various MBE types in improving functional capacity and quality of life (QoL) remain elusively defined. This study aimed to compare the therapeutic efficacy of different MBEs on clinical outcomes in CHF patients using a network meta-analysis (NMA).

**Methods:**

A systematic search was conducted across databases including PubMed, Embase, Web of Science, and the Cochrane Library for randomized controlled trials (RCTs) investigating MBE interventions in CHF, spanning from database inception to January 12, 2026. Two researchers independently performed literature screening, data extraction, and risk of bias assessment (RoB 2.0). R software was utilized for network meta-analysis, SUCRA (Surface Under the Cumulative Ranking Curve) ranking, cluster analysis, and Egger’s test for publication bias. The certainty of evidence was evaluated using the CINeMA (Confidence in Network Meta-Analysis) framework.

**Results:**

Twenty-eight RCTs were ultimately included. The NMA results indicated that Yijinjing was significantly superior to conventional care in improving quality of life (MLHFQ), cardiopulmonary endurance (Peak VO_2_), and cardiac systolic function (LVEF), as well as in reducing cardiac load indicators (NT-proBNP). Furthermore, Yijinjing yielded the highest SUCRA values (0.858–1.000) across these outcomes, demonstrating a high potential intervention probability. Regarding the enhancement of exercise tolerance (6MWD), Meditation showed the highest potential probability (SUCRA = 0.675); however, pairwise comparisons revealed no statistically significant differences between any MBE and conventional care (*p* > 0.05). Cluster analysis further confirmed a synergistic trend of Yijinjing, Liuzijue, and Meditation in enhancing both QoL and exercise endurance. No significant publication bias was detected by Egger’s test for any outcome (*p* > 0.05). The certainty of evidence based on CINeMA was rated as Moderate.

**Conclusion:**

Based on moderate-certainty evidence, Yijinjing shows the highest potential probability for promoting cardiac functional recovery and enhancing quality of life in CHF patients, while meditation particularly of the movement-integrated type shows potential advantages in strengthening functional exercise tolerance. Given the lack of statistical significance in certain indicators (e.g., 6MWD), future intervention designs should focus on the regulatory effects of mind–body integration on kinesiophobia to develop more precise and long-term validated rehabilitation strategies.

**Systematic review registration:**

www.crd.york.ac.uk/prospero, CRD420261308623.

## 1 Introduction

Heart failure is recognized as the common pathway and final clinical manifestation of the progression of various cardiovascular diseases (1, 2). It remains a significant global public health challenge (3, 4). With the aggravation of population aging and the improvement of survival rates after acute myocardial infarction, the prevalence of chronic heart failure (CHF) continues to climb (5, 6). Currently, more than 64 million patients worldwide are troubled by this disease, and its incidence shows a significant upward trend with age (7). The clinical characteristics of chronic heart failure are not only manifested as high hospitalization and mortality rates but also directly lead to a significant decline in patients’ exercise tolerance and severe impairment of quality of life (8, 9). Despite great progress in pharmacological treatment, many patients still experience persistent fatigue and dyspnea after receiving standardized treatment, which limits their daily social functions (10, 11). Therefore, exploring interventions that can effectively enhance functional reserve and improve mental health status has become an urgent need in the current field of cardiovascular medicine.

Mind–body exercise (MBE) is a type of low-to-moderate intensity physical activity that emphasizes the high integration of physical postures, breathing control, and meditative focus, mainly including Tai Chi, Yoga, Qigong, and Pilates (12, 13). Unlike traditional exercises that primarily focus on a single dimension of physical load, mind–body exercises achieve simultaneous intervention in both physiological activation and psychological adjustment through breath regulation and meditative concentration. These exercises feature low risk of injury and high accessibility, as they require no special equipment or venues for implementation, which offers greater practical advantages in terms of safety and convenience. In recent years, as a new type of cardiac rehabilitation means, mind–body exercise has received attention in the field of heart failure rehabilitation due to its regulatory effect on the autonomic nervous system and its gentleness toward cardiac load (14, 15). Existing multiple clinical reviews point out that MBE shows great potential in reducing NT-proBNP levels, improving left ventricular ejection fraction (LVEF), and improving cardiac function grading in patients with chronic heart failure (16, 17). However, the technical characteristics of different schools of mind–body exercise have different focuses; for example, Tai Chi emphasizes the continuity and balance of movements (18), while Yoga focuses on static stretching and deep breathing (19), which may lead to differences in their physiological and psychological benefits.

Although there have been many systematic reviews of single mind–body exercises compared with routine care or traditional exercise in the past, there is still a significant evidence gap in this field, making it difficult to meet the clinical needs of precision rehabilitation. First, most studies are limited to binary comparisons between intervention and control, and very few studies directly conduct head-to-head comparisons between different MBEs (20). This leads to difficulty for clinicians to identify the optimal intervention path for specific rehabilitation goals such as cardiac remodeling or psychological benefits when facing a wide variety of mind–body exercises. Second, previous studies have been relatively singular in the selection of outcome indicators, lacking a comprehensive evaluation of cardiac biomarkers, cardiac function parameters, and clinical functional indicators (21). Therefore, adopting network meta-analysis to rank various MBEs by synthesizing direct and indirect evidence to clarify the relative efficacy positions of different exercise modes is essential to fill the current evidence gap and optimize chronic heart failure rehabilitation guidelines.

This study aims to systematically evaluate and compare the comprehensive effects of various MBEs including Tai Chi, Yoga, Baduanjin, Yijinjing, and Pilates on patients with chronic heart failure through an NMA approach. The research will focus on assessing their relative effectiveness in improving quality of life scores, enhancing exercise tolerance, and optimizing cardiac function indicators. By calculating the probability rankings (P-scores/SUCRA) for each intervention, this study seeks to provide the highest level of evidence-based medical support for the cardiac rehabilitation of CHF patients, assisting clinical decision-makers in formulating more targeted and personalized intervention strategies.

## 2 Materials and methods

The protocol for this study has been registered in PROSPERO (Registration Number: CRD420261308623), defining the primary objectives, inclusion and exclusion criteria, intervention measures, control measures, and planned outcome indicators for assessment. The implementation of this systematic review strictly followed the pre-registered protocol without significant deviation and was conducted and reported in strict accordance with the Preferred Reporting Items for Systematic Reviews and Meta-Analyses (PRISMA 2020) checklist (22).

### 2.1 Search strategy

This study systematically searched PubMed, Embase, CINAHL, Web of Science, Cochrane Library, as well as China National Knowledge Infrastructure (CNKI), Wanfang, and VIP databases, covering the period from database inception to January 12, 2026. The search employed a strategy combining subject headings (e.g., MeSH Terms) and free-text words in titles and abstracts. The logical framework was constructed around four core concepts: (1) heart failure and its clinical classifications (e.g., “Heart Failure,” “HF,” “Cardiac Failure,” “HFpEF,” “HFrEF,” “Cardiac Dysfunction”); (2) mind–body exercise interventions (e.g., “Mind–Body Therapies,” “Tai Chi,” “Baduanjin,” “Eight-Section Brocades,” “Yoga,” “Meditation,” “Mindfulness,” “Qigong,” “Pilates,” “Relaxation Training”); (3) clinical outcome indicators, covering exercise tolerance (e.g., “6-min walk test,” “6MWD”), quality of life (e.g., “Quality of Life,” “MLHFQ”), and cardiac function and biomarkers (e.g., “LVEF,” “Ventricular Function, Left,” “BNP,” “NT-proBNP”); and (4) randomized controlled trials (e.g., “randomized controlled trial,” “RCT,” “placebo,” “random allocation”). Specific search formulas were adjusted according to the specific retrieval rules of different databases. The complete search strings for all databases are provided in the Supplementary material 1. Additionally, we manually searched the reference lists of included articles and related systematic reviews to supplement any omissions.

### 2.2 Inclusion and exclusion criteria

Inclusion and exclusion criteria were strictly formulated according to the PICOS principle. (1) Participants (P) were adult patients with stable chronic heart failure (CHF) meeting clinical diagnostic criteria, with cardiac function usually categorized as NYHA classes I-IV, including subpopulations with preserved (HFpEF) and reduced (HFrEF) ejection fractions; (2) Intervention (I) involved at least one structured mind–body exercise implemented in the experimental group, including Tai Chi, Baduanjin, Liuzijue, Yoga, Pilates, meditation, or relaxation response training, etc.; (3) Comparison (C) involved the control group receiving usual care, simple heart health education, psychological relaxation response, or conventional aerobic/endurance exercise; (4) Outcomes (O) must include at least one key clinical prognostic indicator, such as the Minnesota Living with Heart Failure Questionnaire (MLHFQ), 6-min walk distance (6MWD), peak oxygen uptake (Peak VO_2_), N-terminal pro-B-type natriuretic peptide (NT-proBNP), or left ventricular ejection fraction (LVEF); (5) Study type (S) was limited to publicly published randomized controlled trials (RCTs). Exclusion criteria included: (1) Non-randomized controlled trials (e.g., quasi-experiments, reviews, case reports), duplicate publications, and studies where the intervention did not belong to the category of mind–body exercise; (2) Studies involving patients with severe acute-phase diseases, severe cognitive impairment, or musculoskeletal injuries who were unable to complete exercise interventions; (3) Studies with missing data that could not be obtained by contacting the original authors.

### 2.3 Data collection

Literature screening and data extraction were performed independently by two researchers, with results cross-checked. Discrepancies were resolved through collective discussion or consultation with a third senior researcher. First, all retrieved literature was imported into EndNote management software to remove duplicates. Subsequently, preliminary screening was conducted by reading titles and abstracts, followed by full-text downloads for secondary screening based on inclusion and exclusion criteria to determine the final included studies. Extracted information mainly included: (1) basic information (first author, publication year, country of publication); (2) participant characteristics (sample size, average age, NYHA functional class, ejection fraction subtype) to assess baseline comparability and transitivity between studies; (3) intervention details (specific type of mind–body exercise, such as Tai Chi, Baduanjin, Liuzijue, Yoga, etc.; frequency, duration per session, total intervention period) and specific settings of the control group; (4) core outcome indicators, namely baseline values, endpoint values, or changes (Mean ± SD) for quality of life (QoL, measured by MLHFQ score), 6MWD walking distance, Peak VO_2_, NT-proBNP levels, and LVEF %. For studies providing only standard error (SE) or confidence intervals (CI), standard deviation (SD) conversion was performed according to formulas recommended in the Cochrane Handbook. If key data were missing, attempts were made to contact the original authors to obtain the missing data (23, 24).

### 2.4 Risk of bias and certainty of evidence

The risk of bias in the included studies was independently evaluated by two reviewers using the Cochrane Risk of Bias tool (RoB 2.0) (25). This tool assesses five key domains for randomized controlled trials: bias arising from the randomization process, bias due to deviations from intended interventions, bias due to missing outcome data, bias in measurement of the outcome, and bias in selection of the reported result. The risk of bias for each domain and the overall risk of bias for the study were judged as “Low risk,” “Some concerns,” or “High risk.” For the body of evidence obtained from the network meta-analysis, this study employed the specialized CINeMA (Confidence in Network Meta-Analysis) framework to assess the certainty of evidence for each core outcome indicator (such as MLHFQ score, 6MWD, Peak VO_2_, NT-proBNP, and LVEF %) (26). Evaluation dimensions covered six aspects: within-study bias, reporting bias, indirectness, imprecision, heterogeneity, and incoherence. Finally, the certainty of evidence was categorized into four levels: “High,” “Moderate,” “Low,” or “Very low” based on the comprehensive score. All evaluation tasks were completed independently by two researchers and cross-checked, with discrepancies resolved through joint discussion or consultation with a third senior researcher.

### 2.5 Data analysis

This study utilized the netmeta package in R software (version 4.3.1) to construct random-effects models for network meta-analysis based on the frequentist framework. Before statistical modeling, the transitivity assumption of the evidence network was evaluated by comparing the baseline characteristics of participants across different intervention pathways. For continuous variables such as MLHFQ, 6MWD, Peak VO_2_, NT-proBNP, and LVEF, the standardized mean difference (SMD) and its 95% confidence interval (CI) were uniformly adopted as effect size indicators. First, the netgraph function was used to generate the network evidence plot, where node area represents the total sample size of participants and line thickness represents the number of studies. Second, the Q-test was used to evaluate global consistency of the body of evidence, and the node-splitting method was used to verify local consistency; if *p* > 0.05, the model was considered to meet the consistency assumption. To evaluate the hierarchy of different interventions, this study calculated Surface Under the Cumulative Ranking Curve (SUCRA) values, where higher values (0–100%) represent a greater probability that the intervention is optimal (27). On this basis, this study further employed hierarchical cluster analysis combined with SUCRA values of different outcome indicators to identify intervention clusters with similar therapeutic characteristics across multiple dimensions (28). Finally, a comparison-adjusted funnel plot combined with Egger’s linear regression test was used to assess the presence of small-study effects or significant publication bias, with *p* < 0.05 considered statistically significant.

## 3 Results

### 3.1 Study selection

**Figure 1:**
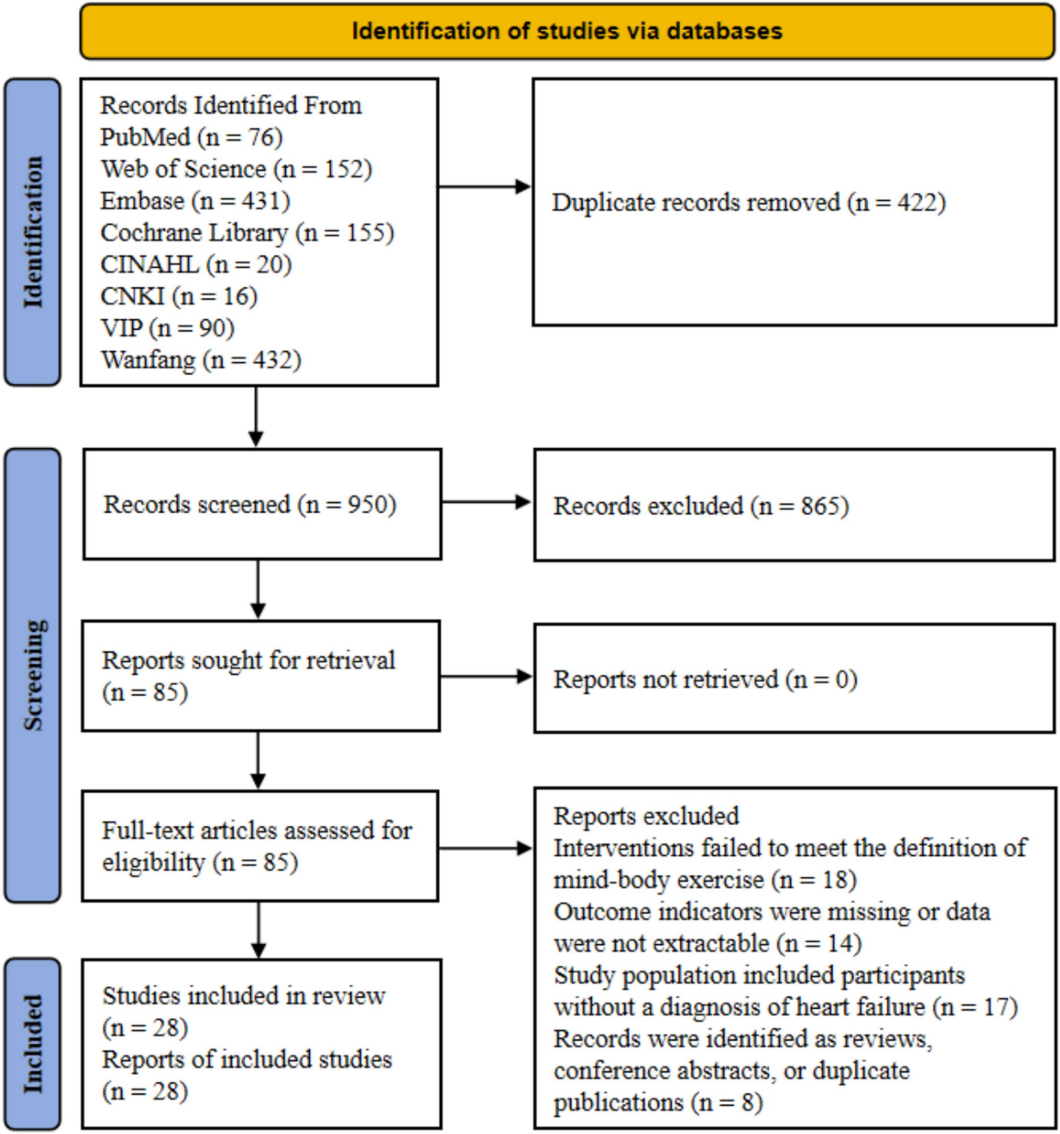
Preferred reporting items for systematic reviews and meta-analysis (PRISMA) study flow diagram.

A total of 1,372 relevant records were identified through the initial database search, leaving 950 records after removing duplicates. During the preliminary screening of titles and abstracts, 865 records were excluded. The primary reasons for exclusion included irrelevant research topics, studies in which participants were not chronic heart failure patients, interventions that did not belong to mind–body exercises (such as pure equipment training or pharmacological interventions), and document types that were reviews or conference abstracts Subsequently, the remaining 85 potentially eligible full-text articles were strictly reviewed. Based on the inclusion and exclusion criteria, 57 articles were excluded, primarily due to inconsistent interventions or the inability to extract key data. Finally, 28 randomized controlled trials were included for the network meta-analysis. The detailed flowchart is shown in Figure 1.

### 3.2 Study characteristics

A total of 28 randomized controlled trials (RCTs) were finally included in this study (29–56), with publication dates spanning from 2004 to 2025 (46, 55) (see Table 1). The geographical distribution is globally diverse, covering countries such as China (29–33, 37–39, 51), the United States (34, 41, 44, 46, 49, 50, 52), Brazil (36, 43), Thailand (35), Italy (47), the United Kingdom (45), Poland (42), India (53), and Sweden (54, 56). The total sample size of participants in the included studies was 1,513, with individual study sample sizes ranging from 16 to 120 (31, 44). Participants were primarily middle-aged and older patients, with mean ages ranging from 52.9 ± 9.4 years to 76.5 years (median) (53, 56). Most participants had cardiac function at NYHA classes II-III, and the research population extensively covered heart failure subtypes such as reduced ejection fraction (HFrEF) (46), mildly reduced ejection fraction (HFmrEF) (33), and preserved ejection fraction (HFpEF) (39).

**Table 1:** Characteristics of the included studies.

First author and year	Country	Sample size (T/C)	Mean age (T/C, Mean ± SD)	Cardiac function (NYHA class)	Heart failure phenotypes	Mind–body exercise type	Frequency/Duration; Total cycle	Control group setting	Core outcomes
Yeh et al. (2004) (46)	USA	15/15	64 ± 13 (Total)	Predominantly Class II	HFrEF (≤40%)	Tai Chi	2 days/week, 60 min; 12 weeks	Combined Intervention	6MWD, MLHFQ, NT-proBNP, Peak VO_2_
Chang et al. (2005) (49)	USA	31/28	66.5 ± 9.2 (Total)	Class II-III	Stable heart failure	Relaxation Response Training	2 sessions/day, 20 min; 15 weeks	Health Education	MLHFQ
Curiati et al. (2005) (43)	Brazil	10/9	74.8 ± 6.7 (Total)	Class II-III	HFrEF (<35%)	Meditation	2 sessions/day, 30 min; 12 weeks	Standard Medical Therapy	MLHFQ
Barrow et al. (2007) (45)	UK	10/10	70.6 ± 10.4/71.3 ± 10.1	Class II-III	HFrEF (<40%)	Tai Chi/Qigong	2 days/week, 60 min; 16 weeks	Standard Medical Therapy	6MWD, MLHFQ
Jayadevappa et al. (2007) (34)	USA	13/10	64.4 ± 5.7/63.8 ± 8.9	Class II-III	HFrEF	Transcendental Meditation	2 sessions/day, 15–20 min; 6 months	Health Education	6MWD, MLHFQ, NT-proBNP
Yeh et al. (2008) (40)	USA	15/15	64 ± 13 (Total)	Predominantly Class II	HFrEF (≤40%)	Tai Chi	2 days/week, 60 min; 12 weeks	Wait-list Control	MLHFQ, 6MWD, NT-proBNP
Pullen et al. (2010) (52)	USA	20/20	57 ± 10/56 ± 11	Class I-III	Mixed type	Yoga	2 days/week, 60 min; 8 weeks	Standard Medical Therapy	Peak VO_2_, MLHFQ
Yeh et al. (2011) (41)	USA	50/50	67 ± 11 (Total)	Class I-III	HFrEF (≤40%)	Tai Chi	2 days/week, 60 min; 12 weeks	Health Education	6MWD, Peak VO_2_, MLHFQ
Caminiti et al. (2011) (47)	Italy	30/30	73.8 ± 6 (Total)	Class II-III	Stable heart failure	Tai Chi + endurance training	3 days/week, 60 min; 12 weeks	Endurance Training	6MWD, MLHFQ, LVEF %
Yeh et al. (2013) (44)	USA	9/7	72.6 ± 7.7/66.6 ± 11.2	Class II-III	HFpEF (≥50%)	Tai Chi	2 days/week, 60 min; 12 weeks	Usual Care	6MWD, MLHFQ, Peak VO_2_
Jain et al. (2014) (53)	India	30/30	52.9 ± 9.4/54.8 ± 8.2	Class I-III	HFrEF (~30%)	Yoga	Daily, 60 min; 12 weeks	Standard Medical Therapy	LVEF %, NT-proBNP, MLHFQ, 6MWD
Drozdz et al. (2015) (42)	Poland	20/20	65.5 ± 11.6/61.2 ± 15	Class II-III	HFrEF (<40%)	Slow breathing training	1 session/day, 30 min; 4 weeks	Standard Medical Therapy	6MWD
Yeh et al. (2016) (50)	USA	50/50	64.5 ± 10 (Total)	Class I-III	HFrEF (≤40%)	Tai Chi	2 days/week, 60 min; 12 weeks	Health Education	6MWD, MLHFQ
Caminiti et al. (2023) (36)	Brazil	16/14	63.3 ± 11.6/59.6 ± 12.3	Class I-III	HFrEF (<40%)	Pilates	2 days/week, 60 min; 16 weeks	Usual Care	6MWD, Peak VO_2_
Zheng et al. (2017) (38)	China	52/52	62.2 ± 7.4/61.8 ± 8.2	Class II-III	HFrEF/HFmrEF	Liuzijue	Daily, 40 min; 3 months	Standard Medical Therapy	6MWD, LVEF %, NT-proBNP, MLHFQ
Hägglund et al. (2017) (54)	Sweden	20/20	64.1 ± 9.4/65.7 ± 8.5	Class I-III	HFrEF/HFpEF	MediYoga	2 days/week, 60 min; 12 weeks	Hydrotherapy	6MWD, NT-proBNP
Chen et al. (2018) (32)	China	30/33	69.1 ± 13.5/71.4 ± 13.7	Class I-II	Mean approximately 56.7%	Baduanjin	3 days/week, 35 min; 12 weeks	Usual Care	MLHFQ
Norman et al. (2018) (56)	Sweden	17/16	76.5/75.0 (Median)	Class II-III	HFrEF/HFpEF	Mindfulness-based intervention	Daily, 30 min; 8 weeks	Standard Medical Therapy	6MWD
Srisoongnern et al. (2021) (35)	Thailand	30/30	66.4 ± 11.2/64.4 ± 10.9	Class II-III	HFmrEF/HFrEF	Walking meditation	2 sessions/day, 30 min; 12 weeks	Walking Training	6MWD, MLHFQ
Ye et al. (2021) (30)	China	40/40	63.2 ± 18.7/65.1 ± 19.9	Class II-III	Not reported	Baduanjin	3–5 days/week, 30 min; 6 months	Combined Intervention	MLHFQ, Peak VO_2_
Ma et al. (2022) (39)	China	50/50	74.6 ± 4.5/74.7 ± 4.7	Class II-III	HFmrEF/HFpEF	Baduanjin + elastic band training	3–5 days/week, 60 min; 12 weeks	Usual Care	6MWD, MLHFQ
Wang et al. (2023) (31)	China	60/60	65.2 ± 9.9/62.6 ± 10.8	Class II-III	Mean approximately 45%	Baduanjin	6 days/week, 30 min; 6 months	Standard Medical Therapy	6MWD, NT-proBNP, LVEF %
Ke (2023) (51)	China	33/33	65.6 ± 8.8/64.8 ± 9.1	Class II-III	HFmrEF (~46%)	Yijinjing	5 days/week, 45 min; 12 weeks	Combined Intervention	6MWD, NT-proBNP, MLHFQ
Cavalcante et al. (2023) (36)	Brazil	13/19	58.0 ± 12.8/54.3 ± 9.8	Class I-II	HFrEF	Meditation and Mindfulness	1 session/week plus daily 45 min; 8 weeks	Usual Care	6MWD, MLHFQ, NT-proBNP
Chen et al. (2023) (37)	China	30/30	60.4 ± 6.0/60.5 ± 6.1	Class II-III	HFmrEF/HFrEF	Liuzijue	5 days/week, 45 min; 12 weeks	Combined Intervention	6MWD, MLHFQ, LVEF %
Ai et al. (2024) (33)	China	28/30	67.7 ± 3.8/68.1 ± 4.8	Class II-IV	HFmrEF/HFpEF	Tai Chi/Baduanjin	3–5 days/week, 30 min; 12 weeks	Usual Care	NT-proBNP, LVEF %, MLHFQ
Li et al. (2024) (29)	China	54/55	60.3 ± 8.8/60.7 ± 9.7	Class I-II	HFmrEF/HFpEF	Modified Baduanjin	3 days/week, 45 min; 12 weeks	Standard Medical Therapy	6MWD, MLHFQ, LVEF %, NT-proBNP
Klompstra et al. (2025) (55)	Sweden	33/36	71 ± 9/70 ± 12	Class II-III	Mixed subtype	Therapeutic Yoga	2 days/week, 60 min; 12 weeks	Usual Care	6MWD, MLHFQ

T: experimental group; C: control group; NYHA: New York Heart Association functional classification; HFrEF: heart failure with reduced ejection fraction (LVEF ≤ 40%); HFmrEF: heart failure with mildly reduced ejection fraction (41–49%); HFpEF: heart failure with preserved ejection fraction (≥ 50%). MLHFQ: Minnesota Living with Heart Failure Questionnaire (lower scores indicate better quality of life); 6MWD (or 6MWT): 6-min walk distance (unit: m), a key indicator for evaluating exercise tolerance; Peak VO_2_: peak oxygen consumption; NT-proBNP: N-terminal pro-B-type natriuretic peptide; LVEF: left ventricular ejection fraction.

The interventions were diverse, primarily categorized into traditional Chinese exercises centered on Tai Chi (33), Baduanjin (32), Liuzijue (37), and Yijinjing (51), as well as mind–body therapies such as Yoga (54), Pilates (47), mindfulness-based interventions (MBI) (36), meditation (43), relaxation training (49), and slow breathing training (42). The intervention frequency ranged from 2 to 5 times per week to daily practice (37, 46), with a single session duration typically of 20–60 min (47, 49), and a total intervention period ranging from 4 weeks to 6 months (34, 42). In addition to conventional pharmacological treatment and health education, some studies in the control groups also introduced walking training (35), hydrotherapy (54), or endurance training (47) as active controls: the walking training intervention had a period of 12 weeks, twice daily, 30 min per session, and consisted of land-based walking training; the hydrotherapy intervention had a period of 12 weeks, twice a week, 45 min per session, and consisted of underwater endurance training; the endurance training intervention had a period of 12 weeks, four times a week, 50 min per session (including 30 min of moderate-intensity aerobic exercise), and consisted of cycling or walking training.

Among the core outcome indicators, quality of life (MLHFQ score) and exercise tolerance (6MWD walking distance) were reported most frequently. Furthermore, some studies reported physiological function indicators such as left ventricular ejection fraction (LVEF %), N-terminal pro-B-type natriuretic peptide (NT-proBNP) levels, and peak oxygen uptake (Peak VO_2_). Additionally, this study systematically evaluated the transitivity assumption of the network meta-analysis by constructing a comparison table of key effect modifiers (Table 1). The results indicated high consistency in the baseline characteristics of participants across different exercise intervention pathways: first, demographically, the average age of each group fell within the core rehabilitation range for older patients of 60–75 years, showing strong population homogeneity; second, clinically, the research subjects at each intervention node were mainly composed of NYHA class II-III patients, and ejection fraction subtypes such as HFrEF were relatively balanced across major intervention groups; third, regarding intervention dosage, although cycles varied from 4 weeks to 6 months, the core intervention period for the vast majority of studies (approximately 80%) was highly concentrated at 8–12 weeks. In summary, the included studies possess good comparability in clinical and methodological characteristics, satisfying the transitivity requirements for conducting a network meta-analysis.

Standard medical therapy: Participants received routine pharmacological treatment or baseline medical management without any additional structured intervention (e.g., routine pharmacotherapy, optimal medical therapy, standard medical therapy, routine baseline treatment, conventional treatment). Usual care: Participants received standard care provided in hospital or community settings, which may include general medical follow-up but no specific exercise or educational program (e.g., usual care, usual community care, standard of care). Health education: Participants attended educational sessions on heart failure management, without any supervised exercise training (e.g., health education, health education group). Combined intervention: Participants received a combination of pharmacological treatment plus additional components such as health education, cardiopulmonary rehabilitation, or exercise (e.g., combined pharmacotherapy and health education, combined pharmacotherapy and cardiopulmonary rehabilitation, combined medication and exercise, medication plus health education). Wait-list control: Participants were placed on a waiting list and received the intervention after the study period (e.g., wait-list control).

### 3.3 Risk of bias

**Figure 2:**
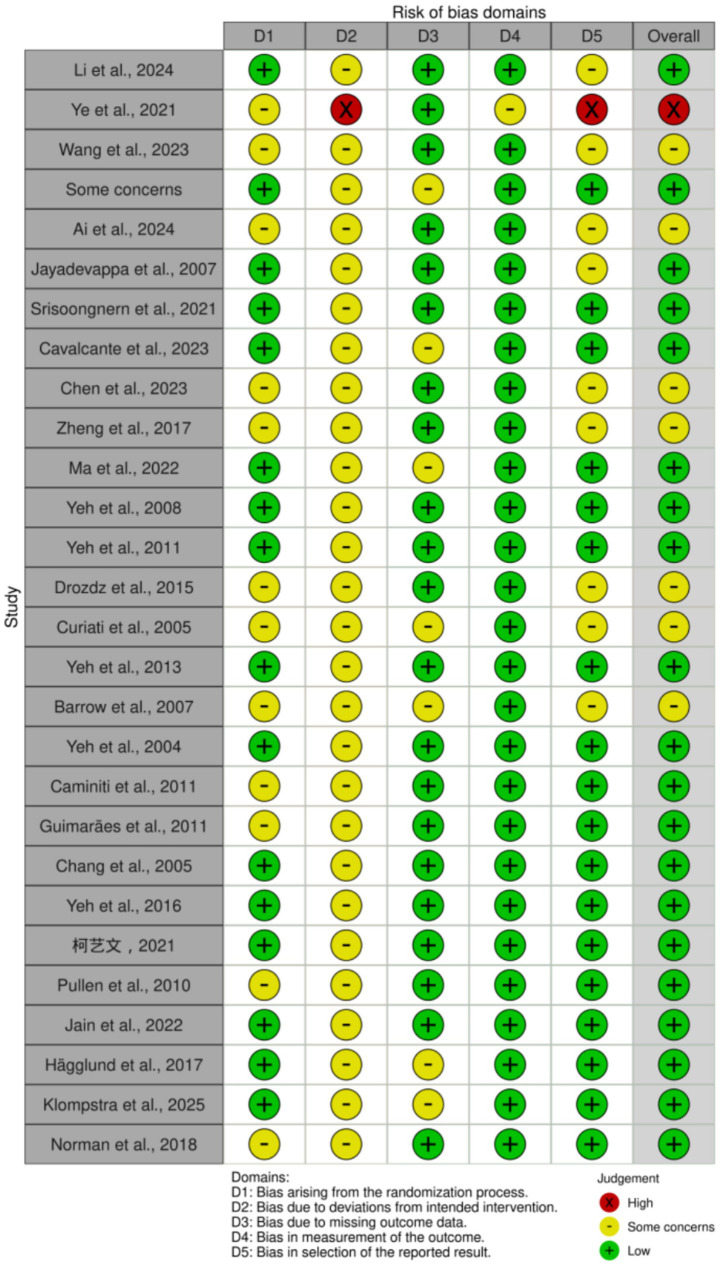
Risk of bias summary: review of the authors judgments about each risk of bias item for each included study.

**Figure 3:**
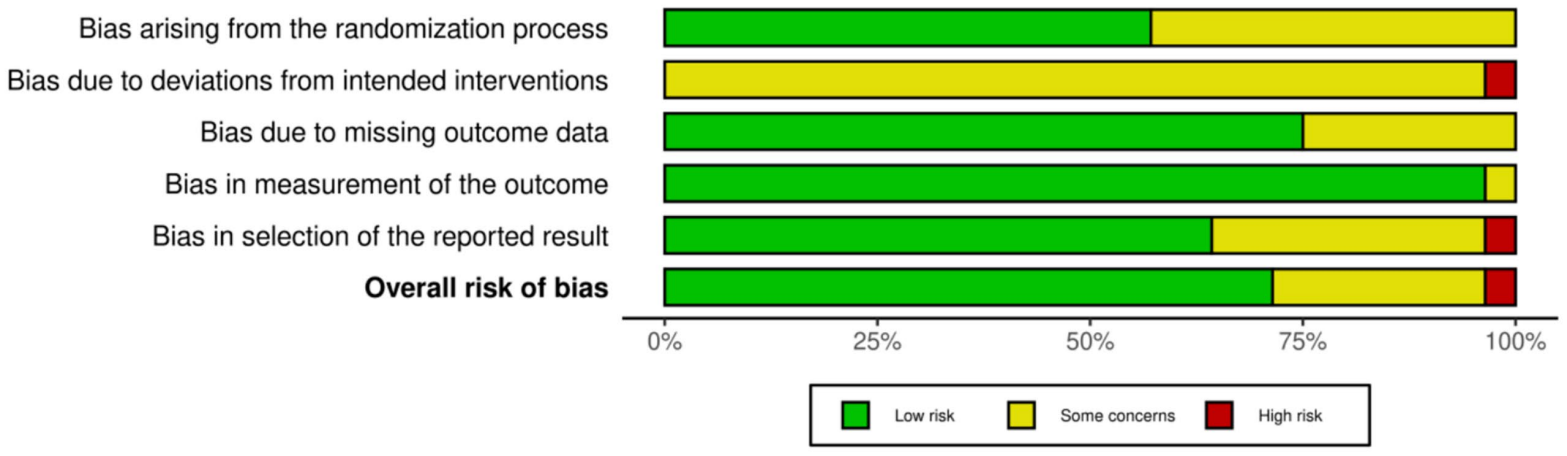
Risk of bias graph: review authors’ judgments about each risk of bias item, presented as percentage of included studies.

The risk of bias for the 28 included studies was rigorously assessed using the RoB 2.0 tool (Figures 2, 3). Regarding the randomization process (D1), 19 studies were judged to be at low risk due to their detailed methodological descriptions. In contrast, 9 studies, including Ye et al. (30) and Wang et al. (31), were rated as having “some concerns” due to insufficient details regarding allocation concealment or random sequence generation. In the domain of deviations from intended interventions (D2), Ye et al. (30) was judged to be at high risk, reflecting significant selection bias during the intervention implementation. While other studies generally faced “some concern” due to the inherent limitations of blinding in non-pharmacological interventions, they minimized potential bias through strict adherence control. For missing outcome data (D3) and selection of the reported result (D5), most studies demonstrated good data integrity, except for Ye et al. (30), which was rated as high risk due to selective reporting. Although some studies [e.g., Barrow et al. (45)] raised concerns regarding the handling of dropouts, the use of recognized objective clinical indicators such as LVEF and NT-proBNP for outcome measurement (D4) effectively bypassed subjective assessor bias, thereby ensuring the high reliability of the primary data.

Overall, the distribution of risk of bias across the 28 studies included 19 at low risk (67.86%), 8 with some concerns (28.57%), and 1 at high risk (3.57%). Taken together, the rigorous study designs and high level of intervention standardization provide a robust evidentiary foundation for this network meta-analysis.

### 3.4 Network evidence characteristics and consistency testing

**Figure 4:**
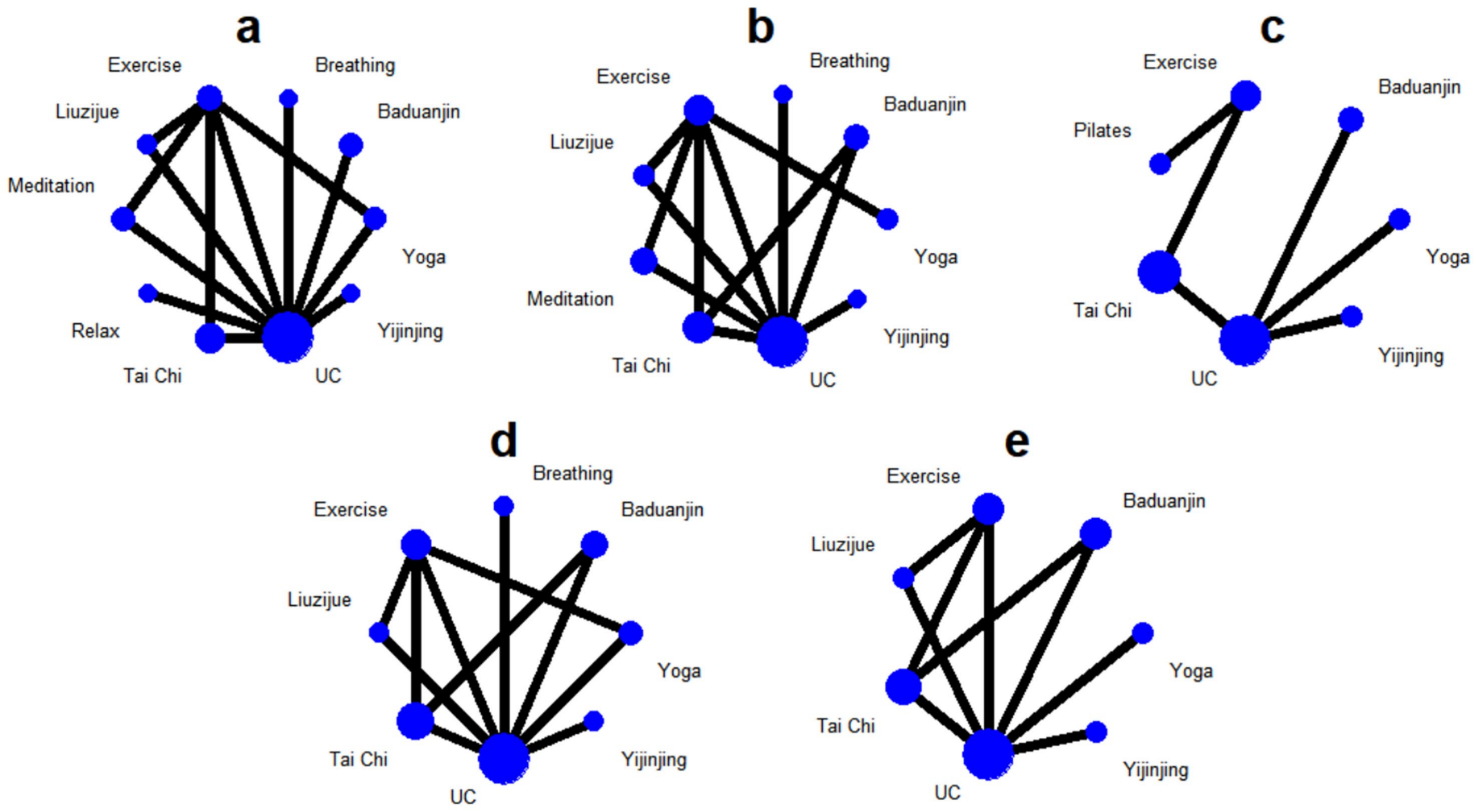
Network evidence plots for the five outcome indicators. **(a)** MLHFQ; **(b)** 6MWD; **(c)** Peak VO_2_; **(d)** NT-proBNP; **(e)** LVEF.

This study constructed network evidence plots for five core outcome indicators (Figure 4), where node size is proportional to the total sample size of the intervention and line thickness reflects the number of studies for direct comparisons. Across all evidence networks, usual care (UC) remained the core node and established direct links with most mind–body exercises, with the network structures for MLHFQ (a) and 6MWD (b) being the densest with more closed loops. To further verify model robustness, this study employed the node-splitting method for local consistency testing (Supplementary Table 1). The analysis showed that for MLHFQ, 6MWD, NT-proBNP, and LVEF, the *p*-values for all intervention pairs for which node-splitting was possible ranged from 0.061 to 0.950 (*p* > 0.05), indicating no significant inconsistency within the closed loops. For the Peak VO_2_ indicator, because its network structure (e.g., the unidirectional connection for Pilates) did not form a closed loop, the model automatically satisfied the consistency assumption structurally. In conclusion, all outcome indicators in this study demonstrated good network connectivity and local consistency, making the use of a consistency model for subsequent data synthesis and efficacy ranking reliable and rigorous.

### 3.5 Network meta-analysis results

#### 3.5.1 Subjective quality of life and exercise tolerance

The NMA results showed that in improving subjective quality of life (MLHFQ), Yijinjing demonstrated a significant advantage (Supplementary Table 2), with an effect size significantly superior to usual care (UC) (SMD = −2.54, 95% CI: −3.62 to −1.46) and all other interventions, including traditional exercise and Tai Chi. Additionally, Liuzijue also showed a significant improvement compared to usual care. Regarding the 6MWD indicator reflecting functional exercise tolerance (Supplementary Table 3), although the SUCRA probability values for Meditation (0.675) and Yijinjing (0.66) were leading, pairwise comparison results showed that the 95% CIs for all interventions relative to usual care included the null value, indicating that given the current statistical power, no statistically significant enhancement of walking distance by mind–body exercise was found. Therefore, the SUCRA rankings for 6MWD should be interpreted as exploratory indicators of potential trends rather than evidence of definitive clinical superiority.

#### 3.5.2 Cardiopulmonary endurance and cardiac physiological function

Synthesis analysis of objective physiological indicators showed that Yijinjing demonstrated potential advantages across multiple dimensions. Regarding Peak VO_2_ (Supplementary Table 4), Yijinjing was the only intervention with statistical significance compared to usual care (SMD = 1.47, 95% CI: 0.34 to 2.61). Analysis of cardiac systolic function (LVEF) (Supplementary Table 5) showed that Yijinjing was not only significantly superior to usual care (SMD = 1.17, 95% CI: 0.57 to 1.77) but also possessed significant statistical advantages in pairwise comparisons with traditional exercise, Tai Chi, and Baduanjin (*p* < 0.05). These findings suggest that Yijinjing has potential clinical value in improving cardiac pumping function in CHF patients.

#### 3.5.3 Cardiac biomarkers

The synthesis results for the cardiac biochemical indicator NT-proBNP are detailed in Supplementary Table 6. In reducing this indicator and alleviating cardiac load, the effect of Yijinjing was significantly superior to usual care (SMD = −2.31, 95% CI: −2.93 to −1.70) and traditional exercise. Meanwhile, Yoga (SMD = −1.18) and Baduanjin (SMD = −0.29) also showed significant statistical significance in reducing NT-proBNP compared to usual care. Overall, the sub-indicator synthesis analysis in this study indicates that Yijinjing is a highly competitive potential intervention choice for CHF patients across multiple rehabilitation dimensions, including subjective quality of life, cardiopulmonary endurance, cardiac physiological function, and biochemical indicators. Furthermore, sensitivity analysis showed that after excluding studies with an intervention period of less than 8 weeks, the efficacy ranking and significance results of pairwise comparisons for the primary outcome indicators remained highly consistent with the original analysis, proving the robustness of the core conclusions.

### 3.6 Efficacy ranking analysis

**Figure 5:**
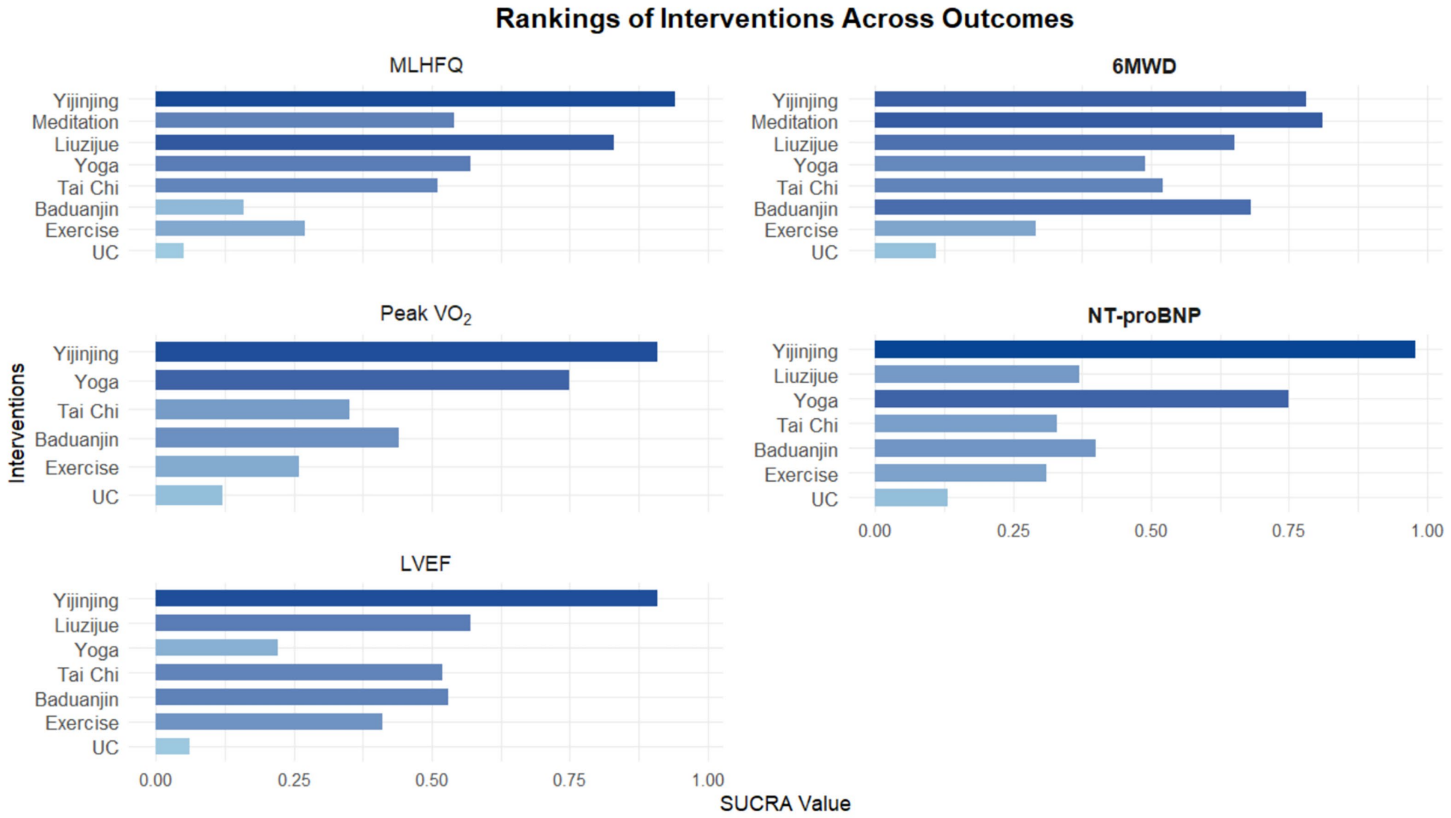
Surface under the cumulative ranking curve (SUCRA) plots for interventions under each outcome indicator.

This study utilized SUCRA values to rank the efficacy of different interventions under the five outcome indicators; a value closer to 1 indicates a higher probability of that intervention being the optimal choice (Figure 5, Supplementary Table 7). Probability analysis results suggested that Yijinjing showed the highest cumulative probability in four key indicators: subjective quality of life (MLHFQ, SUCRA = 0.978), cardiopulmonary endurance (Peak VO_2_, SUCRA = 0.858), cardiac systolic function (LVEF, SUCRA = 0.98), and biomarkers (NT-proBNP, SUCRA = 0.98) (Supplementary Figures 6–10). However, it should be noted that SUCRA rankings represent cumulative probability distributions and do not equate to absolute clinical superiority, particularly for indicators where pairwise comparisons did not reach statistical significance. In the detailed ranking of each indicator, interventions exhibited different clinical advantage characteristics. In the 6MWD dimension, Meditation (SUCRA = 0.675) was slightly higher than Yijinjing (0.66), showing the highest potential probability for increasing walking distance, followed by Baduanjin (0.64) and Liuzijue (0.6). Regarding subjective quality of life improvement, besides Yijinjing, Liuzijue (0.9) also showed a high clinical recommendation probability. For objective physiological indicators, Yoga performed robustly in increasing Peak VO_2_ (0.728) and reducing NT-proBNP (0.831), showing good potential for cardiopulmonary regulation. Notably, traditional exercise (Exercise) and usual care (UC) ranked at the bottom across all outcome indicators. Specifically, the ranking probability for traditional exercise in improving LVEF (0.43) and reducing NT-proBNP (0.479) was significantly lower than most mind–body exercise protocols. It must be emphasized that the SUCRA rankings only represent cumulative probability distributions and do not equate to absolute clinical superiority; particularly for indicators where pairwise comparisons did not reach statistical significance (such as 6MWD), this ranking should be viewed as an exploration of potential trends. These results support, from a probabilistic perspective, that integrating mind–body exercise on the basis of conventional treatment can enhance rehabilitation benefits for CHF patients, with potentially greater clinical value compared to usual care alone or traditional aerobic exercise.

### 3.7 Cluster analysis

**Figure 6:**
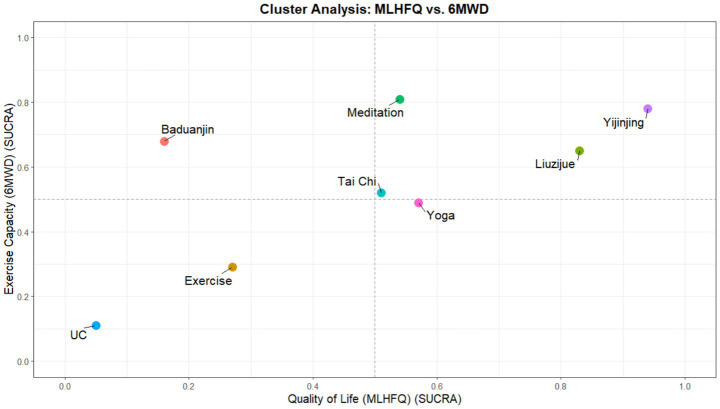
Cluster analysis plot of interventions based on SUCRA values for quality of life (MLHFQ) and exercise tolerance (6MWD).

To further identify the comprehensive benefits of different interventions in improving subjective quality of life (MLHFQ) and functional exercise tolerance (6MWD), this study performed a two-dimensional cluster analysis based on their SUCRA values (Figure 6). The cluster plot was divided into four quadrants using SUCRA = 0.5 as the threshold, aiming to identify the synergistic effects of each intervention across these two core dimensions through spatial distribution. Interventions located in the upper-right quadrant (High QoL & High Exercise Capacity) were considered to have relatively superior comprehensive rehabilitation potential, while those in the lower-left quadrant represented relatively limited performance in both dimensions. The clustering results showed that Yijinjing, Liuzijue, and Meditation were all located in the upper-right quadrant, indicating that these three types of intervention protocols possess potential integrated advantages in chronic heart failure (CHF) rehabilitation. Among them, Yijinjing was positioned closest to the far upper-right of the chart, suggesting a potential high-probability dual-benefit trend in both QoL improvement and exercise tolerance enhancement. Meditation occupied the highest position on the vertical axis (6MWD), showing its optimal potential efficacy in enhancing physical exercise capacity; Liuzijue followed closely behind Yijinjing, reflecting good stability in comprehensive benefits. In contrast, other interventions showed a clearly differentiated distribution. Baduanjin was located in the upper-left quadrant, indicating that while it performed excellently in improving exercise tolerance, its cumulative probability ranking for QoL improvement was relatively low; Yoga and Tai Chi were situated in the lower-right quadrant, showing a characteristic more inclined toward improving patient QoL through psychological regulation. Traditional exercise and Usual Care were located in the lower-left quadrant, forming a distinct spatial cluster with limited efficacy separation from other mind–body exercise protocols. It should be noted that the above cluster distribution is primarily constructed based on SUCRA probability values; given that the 6MWD indicator has not yet achieved statistically significant differences in pairwise comparisons and the overall certainty of evidence is Moderate, the relevant clustering results should be interpreted as potential trends in intervention superiority rather than absolute conclusions. Nevertheless, these findings suggest at the evidentiary level that, compared to traditional rehabilitation methods, mind–body exercises represented by Yijinjing are expected to provide more comprehensive rehabilitation benefits for CHF patients.

**Figure 7:**
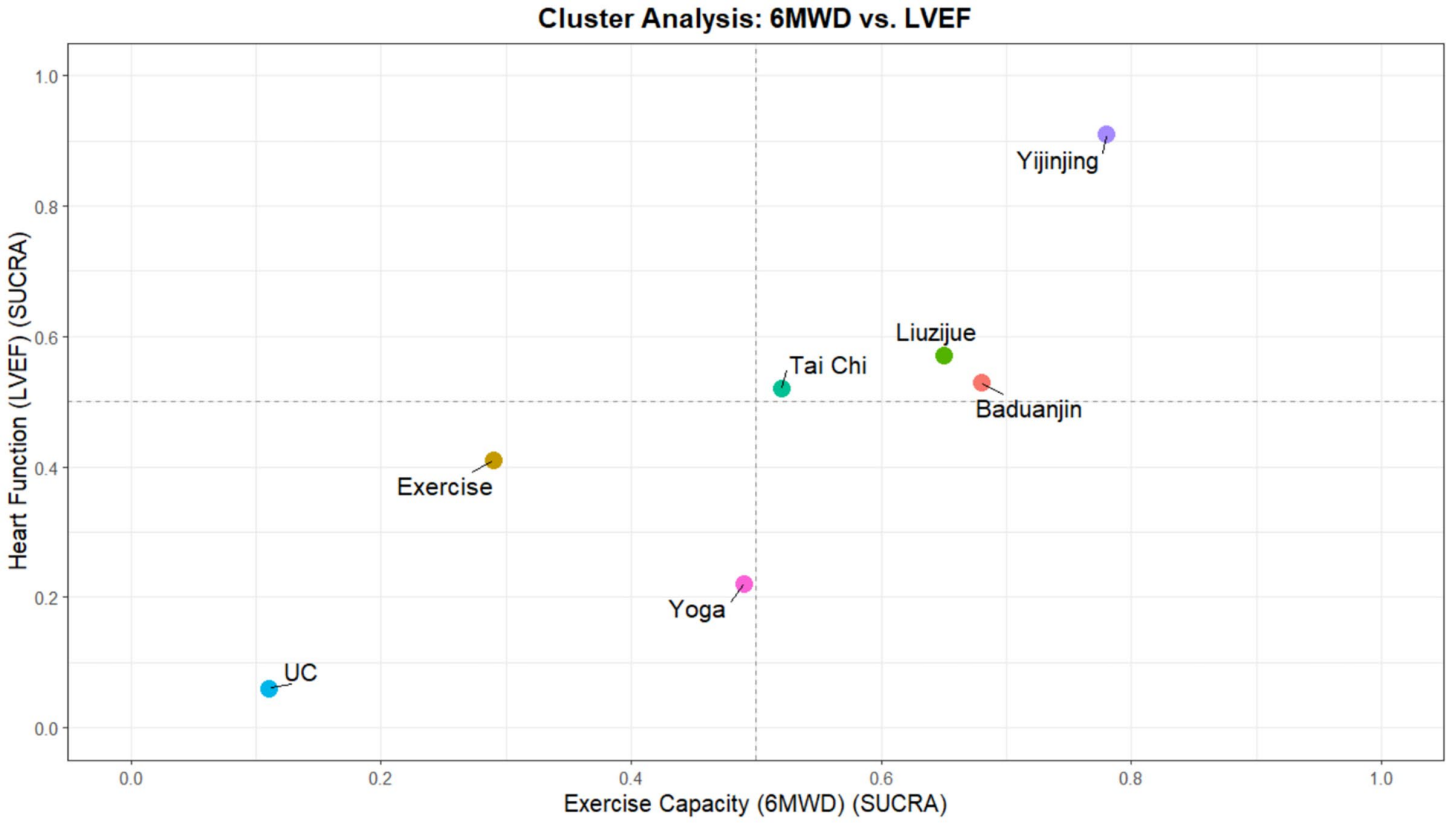
Cluster analysis plot of interventions based on SUCRA values for exercise tolerance (6MWD) and cardiac function (LVEF).

A second dimension of cluster analysis was performed on the SUCRA values of exercise tolerance (6MWD) and cardiac function (LVEF) (Figure 7), aiming to explore the synergistic effects of interventions in enhancing physical performance and improving cardiac physiological structure. This plot similarly used SUCRA = 0.5 as the quadrant divider, with the upper-right quadrant representing the ideal intervention area with a higher probability of clinical benefit in both core dimensions. The results showed that Yijinjing, Liuzijue, and Baduanjin together constituted the core benefit group in the upper-right quadrant. Among them, Yijinjing remained at the extreme position closest to the upper-right corner of the axes; its SUCRA values for both 6MWD and LVEF were close to 0.9, further suggesting its excellent integrative ability in enhancing myocardial contractility and improving systemic exercise endurance. Liuzijue and Baduanjin followed closely, with similar performance in 6MWD, although Liuzijue ranked slightly higher than Baduanjin in the cumulative probability of improving LVEF. In comparison, other intervention protocols exhibited significant functional bias. Yoga was located in the lower area of the cluster plot; although it showed certain potential for improving 6MWD, its cumulative probability for enhancing cardiac physiological function (LVEF) was markedly lower than that of other mind–body exercises. Tai Chi was at the quadrant boundary, exhibiting balanced but moderate benefit characteristics. Traditional exercise (Exercise) and Usual Care (UC) were both deeply entrenched in the lower-left quadrant, showing a vast efficacy gap in spatial distribution compared to the aforementioned mind–body exercise protocols. This finding further supports the clinical recommendation to consider traditional fitness Qigong, such as Yijinjing, as one of the potential exercise protocols for CHF rehabilitation.

### 3.8 Publication bias test

This study systematically assessed the presence of small-study effects or publication bias among the included studies by plotting comparison-adjusted funnel plots and performing Egger’s linear regression test (Supplementary Figures 1–5). The funnel plots showed a relatively symmetrical distribution of study points for the five outcomes, with most studies located near the central axis, suggesting low asymmetry between studies. To further verify publication bias through quantitative means, Egger’s test was implemented for all outcome indicators. The results showed that the *p*-values for Egger’s test were 0.1866 for MLHFQ, 0.9934 for 6MWD, 0.3703 for Peak VO_2_, 0.7889 for NT-proBNP, and 0.4082 for LVEF. Since the p-values for all outcomes all exceeded the statistical threshold of 0.05, there is insufficient evidence to suggest significant publication bias or small-study effects in this study.

### 3.9 Certainty of evidence

This study employed the CINeMA (Confidence in Network Meta-Analysis) framework, specifically designed for NMA, to comprehensively evaluate the reliability of evidence for the five core outcome indicators across six dimensions: within-study bias, reporting bias, indirectness, imprecision, heterogeneity, and incoherence (Table 2). The assessment results showed that the overall certainty of evidence for all five outcomes was judged as “Moderate.” Although the evidence network exhibited good connectivity and local consistency tests found no significant bias (*p* > 0.05), a conservative downgrade was applied in the “within-study bias” dimension because the included studies were mostly non-pharmacological rehabilitation interventions in which blinding of participants and personnel was not feasible, and some indicators (e.g., MLHFQ, NT-proBNP) were influenced by individual high-risk studies [e.g., Ye et al. (30)]. In the evaluation of specific indicators, the core logic for downgrading focused on the precision and robustness of clinical evidence. For quality of life (MLHFQ) and biomarkers (NT-proBNP), although interventions such as Yijinjing showed significant statistical advantages, they were rated as moderate quality due to some potential concerns regarding reporting bias. For physiological function indicators such as 6MWD, Peak VO_2_, and LVEF, downgrading primarily stemmed from “imprecision”: some interventions (e.g., Pilates, Liuzijue) were evaluated in a small number of studies, leading to wide 95% CIs in pairwise comparisons, and none of the intervention pairs for the 6MWD indicator achieved statistical significance compared to usual care, reflecting fluctuations in effect size estimation in the current body of evidence. In summary, while existing evidence supports the efficacy ranking of Yijinjing, the conclusions should be interpreted prudently within a moderate-certainty evidence framework, considering study limitations and measurement uncertainties.

**Table 2:** Summary of certainty of evidence for network meta-analysis based on the CINeMA framework.

Outcome	Within-study bias	Reporting bias	Indirectness	Imprecision	Heterogeneity	Inconsistency	Confidence level
MLHFQ	Some concerns 1	Some concerns	Low	Low	Low	Low	Moderate
6MWD	Low	Low	Low	Major concerns 2	Low	Low	Moderate
Peak VO_2_	Low	Low	Some concerns	Some concerns	Low	Low	Moderate
NT-proBNP	Some concerns 1	Some concerns	Low	Low	Low	Low	Moderate
LVEF	Low	Low	Low	Some concerns 2	Some concerns	Low	Moderate

1. Within-study bias: Primarily due to the inherent inability to implement double-blinding in non-pharmacological interventions and the presence of individual studies [e.g., Ye et al. (30)] judged as being at high risk of bias. 2. Imprecision: Arising from wide confidence intervals caused by small sample sizes for certain interventions, or effect sizes failing to reach statistical significance (e.g., for 6MWD).

## 4 Discussion

### 4.1 Study summary

Through a network meta-analysis of 28 randomized controlled trials, this study systematically evaluated the effects of various mind–body exercises (MBE) on clinical outcomes in patients with chronic heart failure (CHF). The research found that Yijinjing demonstrated a significant and comprehensive advantage in improving both subjective and objective rehabilitation indicators in heart failure patients. In the synthesis of therapeutic effects, Yijinjing was significantly superior to usual care across four key dimensions: subjective quality of life (MLHFQ), objective cardiopulmonary endurance (Peak VO_2_), cardiac pumping function (LVEF), and biochemical markers of cardiac load (NT-proBNP). Furthermore, its SUCRA values (0.858–1.000) consistently ranked first among the aforementioned indicators. This chain of evidence suggests that traditional fitness Qigong, represented by Yijinjing, can not only improve patients’ self-perception but also substantially promote the repair and remodeling of cardiac function at the physiological and biochemical levels.

In the horizontal comparison of multi-dimensional rehabilitation benefits, this study further identified the functional predispositions of different mind–body exercises through cluster analysis. The results showed that Yijinjing, Liuzijue, and Meditation exhibited a high degree of synergistic effect in enhancing quality of life and exercise tolerance, collectively occupying the optimal benefit quadrant of the cluster plot. Although the exercise tolerance (6MWD) indicator did not show statistically significant differences for any intervention compared to usual care, the point estimates for Yijinjing and Meditation still displayed a leading positive trend. Consequently, the high SUCRA rankings for Meditation and Yijinjing in this dimension reflect probability distributions only and should not be construed as establishing clinical dominance in the absence of statistically significant pairwise comparisons. Additionally, Yoga and Baduanjin demonstrated reliable biochemical benefits in reducing NT-proBNP levels. Together with the CINeMA certainty of evidence evaluation and Egger’s test results, the efficacy ranking in this study possesses good robustness and can provide reliable evidence-based support for the formulation of clinical exercise prescriptions for patients with chronic heart failure.

### 4.2 Exploration of efficacy ranking and potential mechanisms

The results of this study present both similarities and differences compared to previous research in related fields. Regarding the fundamental conclusion that the rehabilitation benefits of mind–body exercise (MBE) for chronic heart failure patients are superior to usual care, this study converges with the results of most published meta-analyses (21, 57). A meta-analysis focusing on cardiac patients further confirmed that systematic physical activity can significantly enhance left ventricular ejection fraction (LVEF), peak oxygen uptake (Peak VO_2_), and quality of life, providing solid empirical support for the core position of exercise intervention in cardiac rehabilitation (58). However, in terms of the efficacy ranking of specific intervention protocols, previous studies mostly emphasized the clinical benefits of Tai Chi or Baduanjin, whereas this study suggests that Yijinjing may demonstrate promising efficacy across multiple cardiac function indicators. The reason for this discrepancy may lie in the inclusion of high-quality randomized controlled trials (RCTs) conducted on Yijinjing in recent years and the utilization of network meta-analysis to achieve indirect comparisons between different interventions. Furthermore, past studies rarely differentiated Buddhist walking meditation from broad psychological interventions, while this study explored the potential role of this meditation modality when integrated with physical activity in enhancing physical exercise tolerance through categorical definition.

According to the NMA results, Yijinjing ranked first in the SUCRA rankings for LVEF, Peak VO_2_, and NT-proBNP. This trend may theoretically reflect the movement characteristics of Yijinjing and its potential regulatory effects on cardiopulmonary interaction. Yijinjing involves extensive spinal rotation and thoracic expansion movements and emphasizes the coordination of diaphragmatic breathing; based on this, it is speculated that this might help enhance vagal tone and inhibit the overactivation of the sympathetic nervous system (59). This physiological remodeling effect was also corroborated in the study by Gupta et al. (58), which stated that regular exercise directly improves cardiac pumping function and stroke volume by optimizing cardiopulmonary parameters (60). According to existing exercise physiology hypotheses, this autonomic regulation may improve myocardial contractility in patients and reduce cardiac afterload (61). Additionally, the unique isometric contraction and eccentric control of Yijinjing may stimulate the skeletal muscles of the lower limbs, thereby promoting peripheral vasodilation and improving mitochondrial function in muscles (62), suggesting its biological hypothetical basis for peripheral muscle metabolic adaptation in enhancing peak oxygen uptake (63). Regarding the 6MWD indicator, this study categorized interventions such as Buddhist walking meditation and mindfulness as meditation types, and their SUCRA values showed the highest probability of benefit among all interventions. This result may be attributed to the influence of combined physical activity and psychological regulation on functional exercise tolerance (64). Since chronic heart failure patients often suffer from kinesiophobia, the mindfulness elements in Buddhist walking meditation may help regulate patients’ catastrophizing cognition regarding dyspnea (35). This mechanism of improving psychological state through exercise and subsequently enhancing quality of life is also reflected in other chronic disease fields. For example, research by Gupta et al. on breast cancer patients and the overall cancer population indicated that regular exercise intervention can significantly improve patients’ health-related quality of life. Although the disease backgrounds differ, the core rehabilitation logic is consistent, namely that exercise intervention can effectively alleviate the dual physical and mental pressure brought by chronic diseases (65). By reducing psychological stress and subjective perception of effort during exercise, this intervention modality involving walking training may optimize gait efficiency without increasing cardiac burden (36). Although the league table results for 6MWD did not show statistical differences, this ranking trend suggests that for heart failure populations characterized by kinesiophobia, introducing meditation training integrated with physical activity may have potential rehabilitation value (34). It should be clarified that the aforementioned explanations regarding autonomic regulation and cardiac physiological remodeling currently remain theoretical speculations and require more specific mechanism trials in chronic heart failure (CHF) populations for confirmation. From the spatial distribution of cluster analysis, Yijinjing, Liuzijue, and meditation are all located within the benefit quadrant for improving quality of life and exercise tolerance, showing multi-dimensional synergistic effects. This distribution characteristic may mean that the advantage of mind–body exercise lies in the overall effect achieved through respiratory regulation, cognitive restructuring, and physical training (66). In terms of evidence robustness, Egger’s test results showed no significant publication bias for any outcome indicator. Although the CINeMA evaluation judged the certainty of evidence as moderate due to the blinding limitations of non-pharmacological interventions and the influence of individual study risks, sensitivity analysis showed that the structure of the overall evidence network remained unshaken. These findings may provide a reference for formulating personalized rehabilitation prescriptions for chronic heart failure patients based on physiological function repair and behavioral capacity enhancement.

The potential efficacy of mind–body exercise (MBE) observed in this network meta-analysis may be rooted in its distinct “top-down” neuro-physiological integration mechanisms, which fundamentally differ from the “bottom-up” peripheral adaptation pathways characteristic of conventional aerobic and resistance training. Specifically, MBE (particularly Yijinjing and Tai Chi) emphasizes the conscious integration of diaphragmatic breathing, spinal rotation, and meditative focus, which collectively enhance vagal tone and inhibit sympathetic overactivation—a mechanism supported by Russo et al.’s work on slow breathing physiology (59). This autonomic rebalancing is particularly relevant for CHF patients, as it reduces cardiac afterload and improves heart rate variability without imposing excessive hemodynamic stress. Furthermore, the isometric and eccentric control components inherent in MBE stimulate skeletal muscle metaboreceptors, promoting peripheral vasodilation and mitochondrial biogenesis through PGC-1α signaling pathways (63). Notably, the meditative elements in MBE may specifically target kinesiophobia and catastrophizing cognition regarding dyspnea, as evidenced by the clustering results showing meditation’s advantage in 6MWD (12), thereby optimizing exercise tolerance via psychological pathways that pure physical training cannot access.

In contrast, traditional aerobic training primarily enhances functional capacity through peripheral metabolic adaptations—specifically, increased oxidative enzyme activity, capillary density, and mitochondrial volume in skeletal muscles—consistent with Coats’ “muscle hypothesis” of heart failure (62). While these adaptations improve oxygen extraction and reduce ventilatory drive, aerobic exercise simultaneously imposes significant volume and pressure loads on the failing heart. Resistance training, conversely, predominantly targets muscular strength through hypertrophic and neuromuscular adaptations but offers limited direct modulation of autonomic tone or baroreceptor sensitivity, as demonstrated in comparative studies of exercise modalities in cardiac populations. Therefore, MBE appears to occupy a unique mechanistic niche: it delivers comparable peripheral metabolic benefits (as evidenced by improvements in Peak VO ~ 2 ~ and 6MWD) while simultaneously conferring additional cardioprotective effects through enhanced parasympathetic modulation and reduced neurohormonal activation (lower NT-proBNP) (64). This dual-action mechanism—combining physiological remodeling with psychological resilience—may explain why Yijinjing showed high SUCRA rankings across both objective cardiac function indicators and subjective quality of life measures.

### 4.3 Study limitations

Although this study followed strict systematic review and network meta-analysis standards, several potential limitations remain. First, the risk of bias assessment for the included studies indicated that it was difficult to achieve double-blinding of participants and personnel during implementation. Since mind–body exercise is a non-pharmacological rehabilitation method, the participants’ awareness of intervention allocation may, to some extent, influence the evaluation of subjective outcome indicators, particularly the MLHFQ quality of life scale, potentially leading to an overestimation of effect sizes. Although this study marked this risk in the CINeMA evaluation, this inherent methodological limitation may still affect the certainty of evidence. Second, there is some heterogeneity in the intervention “dosage” of different mind–body exercise protocols. The included studies were not fully unified in terms of intervention frequency, which ranged from twice weekly to daily practice; duration, which varied from 8 to 24 weeks; and the intensity of a single session. This variation in protocol design may be a potential source of heterogeneity and could affect the robustness of the relative efficacy rankings between different interventions. Furthermore, although this study primarily targeted chronic heart failure patients, a strict stratified analysis for heart failure with preserved ejection fraction (HFpEF) and chronic heart failure with reduced ejection fraction (HFrEF) was not performed, which might limit the precise application of the conclusions in specific subgroup populations.

Third, some comparison paths in the evidence network remain thin. Despite the good overall connectivity of the evidence network, the number of original RCTs for specific interventions such as Yijinjing and Buddhist walking meditation is relatively small, and some results rely primarily on indirect comparisons. This may lead to wide 95% CIs for indicators such as 6MWD in the league table, thereby weakening statistical significance. Additionally, most current studies have short follow-up periods and lack in-depth tracking of the impact of mind–body exercise on long-term prognosis such as all-cause mortality and rehospitalization rates; the long-term adherence and long-term rehabilitation effects of mind–body exercise still need further verification in future studies. Finally, the geographical distribution of the included studies may have certain limitations. Since mind–body exercises such as Qigong and Tai Chi have deep cultural backgrounds, high-quality clinical trials are currently concentrated in Asia. This concentration of regional distribution may challenge the generalizability of the research conclusions across different cultural backgrounds or ethnic groups. Future research should encourage international collaborative studies with multi-center, large-sample, and long-term follow-up designs to further clarify the application value of different mind–body exercise protocols in the global rehabilitation practice of chronic heart failure.

### 4.4 Practical implications

The results of this study provide an evidence-based foundation for optimizing cardiac rehabilitation protocols for patients with chronic heart failure. Traditional rehabilitation models usually center on aerobic exercise or resistance training; however, this study confirmed through network meta-analysis that mind–body exercises represented by Yijinjing may offer potential advantages in improving cardiac physiological remodeling and functional indicators. This suggests that when formulating rehabilitation guidelines, medical institutions could consider incorporating structured mind–body exercises as a vital supplement or alternative to traditional exercise rehabilitation to enhance overall rehabilitation benefits.

Regarding the specific rehabilitation needs of patients, this study offers differentiated intervention options. For patients whose primary goals are to increase left ventricular ejection fraction, reduce NT-proBNP levels, and enhance cardiopulmonary endurance, Yijinjing may be considered as a potential exercise intervention. For patients with limited functional exercise tolerance or those characterized by significant kinesiophobia, clinicians may prioritize the introduction of meditation-based interventions including Buddhist walking meditation, utilizing its dual psychosomatic regulatory features to improve walking efficiency and activity performance. This evidence-based matching of interventions to patient needs assists clinicians in formulating more targeted and personalized exercise prescriptions. Mind–body exercise demonstrates high feasibility and cost-effectiveness in clinical practice. Such interventions have low requirements for venues and equipment, and their low-to-moderate intensity exercise characteristics are suitable for long-term practice by patients with different NYHA cardiac functional classes. Compared to physical rehabilitation methods that rely on expensive equipment, promoting mind–body exercise helps alleviate the economic burden on patients and may improve exercise adherence after hospital discharge. In future chronic disease management systems, protocols such as Yijinjing, Liuzijue, and meditation are expected to become essential tools for community-based and home-based cardiac rehabilitation.

## 5 Conclusion

The results of this network meta-analysis indicate that integrating mind–body exercise on the basis of conventional treatment holds potential improvement value for the functional capacity and quality of life of patients with chronic heart failure (CHF). Evaluation results based on the CINeMA framework show that the evidence for related outcome indicators generally remains at a moderate-certainty level. Probability ranking results indicate that Yijinjing exhibits a high potential probability of benefit in enhancing cardiac systolic function (LVEF), cardiopulmonary endurance (Peak VO_2_), and quality of life (MLHFQ). This suggests that Yijinjing has certain application prospects in promoting cardiac physiological remodeling and functional recovery; however, its definitive efficacy remains limited by the certainty of existing evidence. Regarding exercise tolerance (6MWD), meditation-based interventions including Buddhist walking meditation show a high probability of benefit; this may reflect the potential contribution of psychosomatic regulatory mechanisms in alleviating kinesiophobia and enhancing walking efficiency. In clinical applications, physicians may select intervention protocols according to the specific rehabilitation needs of the patient. Given that the current quality of evidence is rated as moderate and the statistical significance of certain indicators such as 6MWD has not yet been clarified, Yijinjing should not be regarded as the sole “core” or “preferred” protocol. The long-term rehabilitation effects, clinical promotion value, and deep physiological mechanisms of mind–body exercise still require further exploration through more randomized controlled trials with large sample sizes, high transparency, and mechanism-validation designs in the future.

## Data Availability

The original contributions presented in the study are included in the article/Supplementary material, further inquiries can be directed to the corresponding author.
