# Supplementary material for: Comparative efficacy of different mind–body exercises on functional capacity and quality of life in patients with chronic heart failure: a systematic review and network meta-analysis
Source: Front Public Health. 2026 May 1;14:1802127. doi: 10.3389/fpubh.2026.1802127 (PMC13184644; doi:10.3389/fpubh.2026.1802127)
Supplement: Supplementary file 1 [file Table_1.DOCX]

Table 1 Summary of local consistency test based on the node-splitting method

| **Outcomes** | **Comparison** | **TE** | **seTE** | **Statistic** | **P** |
| --- | --- | --- | --- | --- | --- |
| MLHFQ | Exercise vs. Liuzijue | -0.21 | 0.927 | -0.227 | 0.821 |
|  | Exercise vs. Meditation | -0.642 | 0.718 | -0.893 | 0.372 |
|  | Exercise vs. Tai Chi | 0.503 | 0.602 | 0.836 | 0.403 |
|  | Exercise vs. UC | 0.671 | 0.754 | 0.89 | 0.374 |
|  | Exercise vs. Yoga | -0.403 | 0.72 | -0.56 | 0.576 |
|  | Liuzijue vs. UC | 0.627 | 1.407 | 0.446 | 0.656 |
| 6MWD | Baduanjin vs. Tai Chi | -0.642 | 1.192 | -0.539 | 0.59 |
|  | Baduanjin vs. UC | -2.043 | 1.929 | -1.059 | 0.289 |
|  | Exercise vs. Liuzijue | -0.18 | 1.552 | -0.116 | 0.908 |
|  | Exercise vs. Meditation | 1.043 | 1.265 | 0.824 | 0.41 |
|  | Exercise vs. Tai Chi | -0.394 | 1.104 | -0.357 | 0.721 |
|  | Exercise vs. UC | -0.647 | 1.239 | -0.523 | 0.601 |
|  | Liuzijue vs. UC | -0.899 | 2.312 | -0.389 | 0.697 |
|  | Tai Chi vs. UC | -1.343 | 1.005 | -1.336 | 0.181 |
| Peak VO2​ | 所有干预对 | — | — | — | N/A* |
| NT-proBNP | Baduanjin vs. Tai Chi | 0.116 | 0.338 | 0.345 | 0.73 |
|  | Baduanjin vs. UC | 0.099 | 0.589 | 0.168 | 0.867 |
|  | Exercise vs. Liuzijue | 0.154 | 1.076 | 0.143 | 0.886 |
|  | Exercise vs. Tai Chi | 0.637 | 0.437 | 1.458 | 0.145 |
|  | Exercise vs. Yoga | -0.981 | 0.523 | -1.877 | 0.061 |
|  | Tai Chi vs. UC | 0.611 | 0.364 | 1.681 | 0.093 |
| LVEF | Baduanjin vs. Tai Chi | -0.513 | 0.427 | -1.202 | 0.229 |
|  | Baduanjin vs. UC | 0.435 | 0.754 | 0.577 | 0.564 |
|  | Exercise vs. Tai Chi | 0.726 | 0.632 | 1.149 | 0.251 |
|  | Tai Chi vs. UC | -0.031 | 0.491 | -0.063 | 0.95 |

Table 2 League table of network meta-analysis for the effects of different mind-body exercises on quality of life (MLHFQ)

| **Interventions** | **Yijinjing** | **Liuzijue** | **TaiChi** | **Yoga** | **Meditation** | **Exercise** | **Breathing** | **Baduanjin** | **Relax** | **Usual care** |
| --- | --- | --- | --- | --- | --- | --- | --- | --- | --- | --- |
| Yijinjing | Yijinjing | . | . | . | . | . | . | . | . | -2.54 (-3.62; -1.46) |
| Liuzijue | -0.63 (-1.98; 0.72) | Liuzijue | . | . | . | -1.20 (-2.54; 0.13) | . | . | . | -1.85 (-2.70; -1.01) |
| Tai Chi | -1.54 (-2.70; -0.37) | -0.91 (-1.80; -0.02) | Tai Chi | . | . | -0.64 (-1.44; 0.17) | . | . | . | -0.92 (-1.40; -0.45) |
| Yoga | -1.63 (-2.89; -0.37) | -1.00 (-2.00; 0.00) | -0.09 (-0.83; 0.64) | Yoga | . | -0.11 (-1.11; 0.90) | . | . | . | -1.03 (-1.78; -0.27) |
| Meditation | -1.73 (-2.98; -0.49) | -1.11 (-2.09; -0.12) | -0.20 (-0.91; 0.51) | -0.11 (-0.95; 0.73) | Meditation | 0.14 (-0.90; 1.18) | . | . | . | -0.96 (-1.67; -0.26) |
| Exercise | -1.94 (-3.16; -0.72) | -1.31 (-2.22; -0.41) | -0.41 (-0.99; 0.18) | -0.31 (-1.02; 0.39) | -0.21 (-0.91; 0.49) | Exercise | . | . | . | -0.05 (-1.39; 1.29) |
| Breathing | -2.07 (-3.58; -0.56) | -1.44 (-2.77; -0.11) | -0.53 (-1.67; 0.61) | -0.44 (-1.67; 0.80) | -0.33 (-1.55; 0.89) | -0.12 (-1.32; 1.07) | Breathing | . | . | -0.47 (-1.53; 0.58) |
| Baduanjin | -2.14 (-3.33; -0.95) | -1.51 (-2.45; -0.57) | -0.60 (-1.25; 0.05) | -0.51 (-1.31; 0.29) | -0.41 (-1.18; 0.37) | -0.20 (-0.95; 0.55) | -0.07 (-1.23; 1.08) | Baduanjin | . | -0.40 (-0.88; 0.09) |
| Relax | -2.35 (-3.81; -0.89) | -1.72 (-2.99; -0.45) | -0.81 (-1.88; 0.26) | -0.72 (-1.89; 0.45) | -0.61 (-1.76; 0.54) | -0.41 (-1.54; 0.73) | -0.28 (-1.72; 1.15) | -0.21 (-1.30; 0.88) | Relax | -0.19 (-1.17; 0.79) |
| Usual care | -2.54 (-3.62; -1.46) | -1.91 (-2.72; -1.11) | -1.00 (-1.44; -0.57) | -0.91 (-1.55; -0.27) | -0.80 (-1.41; -0.20) | -0.60 (-1.17; -0.03) | -0.47 (-1.53; 0.58) | -0.40 (-0.88; 0.09) | -0.19 (-1.17; 0.79) | Usual care |

Table 3 League table of network meta-analysis for the effects of different mind-body exercises on exercise tolerance (6MWD)

| **Interventions** | **Meditation** | **Yijinjing** | **Baduanjin** | **Liuzijue** | **TaiChi** | **Yoga** | **Breathing** | **Exercise** | **Usual care** |
| --- | --- | --- | --- | --- | --- | --- | --- | --- | --- |
| Meditation | Meditation | . | . | . | . | . | . | -0.05 (-1.94; 1.85) | 0.98 (-0.02; 1.98) |
| Yijinjing | -0.13 (-2.22; 1.97) | Yijinjing | . | . | . | . | . | . | 0.93 (-0.95; 2.82) |
| Baduanjin | 0.06 (-1.22; 1.34) | 0.19 (-1.90; 2.28) | Baduanjin | . | -0.15 (-2.04; 1.74) | . | . | . | 0.62 (-0.31; 1.56) |
| Liuzijue | 0.11 (-1.46; 1.68) | 0.23 (-2.07; 2.53) | 0.04 (-1.55; 1.64) | Liuzijue | . | . | . | 0.54 (-1.51; 2.60) | 0.62 (-0.78; 2.01) |
| Tai Chi | 0.33 (-0.81; 1.47) | 0.45 (-1.58; 2.49) | 0.27 (-0.84; 1.38) | 0.22 (-1.26; 1.70) | Tai Chi | . | . | 0.40 (-0.99; 1.79) | 0.23 (-0.62; 1.08) |
| Yoga | 0.37 (-1.45; 2.20) | 0.50 (-2.06; 3.06) | 0.31 (-1.62; 2.24) | 0.27 (-1.77; 2.30) | 0.05 (-1.68; 1.77) | Yoga | . | 0.19 (-1.17; 1.55) | . |
| Breathing | 0.59 (-1.53; 2.70) | 0.71 (-1.96; 3.39) | 0.53 (-1.58; 2.64) | 0.48 (-1.84; 2.80) | 0.26 (-1.79; 2.31) | 0.21 (-2.36; 2.79) | Breathing | . | 0.22 (-1.68; 2.13) |
| Exercise | 0.57 (-0.65; 1.79) | 0.69 (-1.48; 2.86) | 0.51 (-0.87; 1.88) | 0.46 (-1.05; 1.98) | 0.24 (-0.83; 1.30) | 0.19 (-1.17; 1.55) | -0.02 (-2.21; 2.16) | Exercise | -0.23 (-2.31; 1.85) |
| Usual care | 0.81 (-0.11; 1.73) | 0.93 (-0.95; 2.82) | 0.75 (-0.16; 1.65) | 0.70 (-0.62; 2.03) | 0.48 (-0.29; 1.25) | 0.43 (-1.30; 2.17) | 0.22 (-1.68; 2.13) | 0.24 (-0.83; 1.32) | Usual care |

Table 4 League table of network meta-analysis for the effects of different mind-body exercises on cardiorespiratory fitness (Peak VO_2_)

| **Interventions** | **Yijinjing** | **Yoga** | **Pilates** | **Baduanjin** | **TaiChi** | **Exercise** | **Usual care** |
| --- | --- | --- | --- | --- | --- | --- | --- |
| Yijinjing | Yijinjing | . | . | . | . | . | 1.47 (0.34; 2.61) |
| Yoga | 0.36 (-1.32; 2.04) | Yoga | . | . | . | . | 1.11 (-0.12; 2.34) |
| Pilates | 0.53 (-1.57; 2.64) | 0.17 (-1.99; 2.33) | Pilates | . | . | 0.90 (-0.53; 2.34) | . |
| Baduanjin | 0.78 (-0.60; 2.15) | 0.42 (-1.04; 1.87) | 0.24 (-1.69; 2.18) | Baduanjin | . | . | 0.70 (-0.07; 1.47) |
| Tai Chi | 1.21 (-0.06; 2.48) | 0.85 (-0.51; 2.21) | 0.68 (-1.00; 2.36) | 0.43 (-0.53; 1.39) | Tai Chi | 0.23 (-0.65; 1.10) | 0.26 (-0.31; 0.84) |
| Exercise | 1.44 (-0.11; 2.98) | 1.08 (-0.54; 2.69) | 0.90 (-0.53; 2.34) | 0.66 (-0.64; 1.96) | 0.23 (-0.65; 1.10) | Exercise | . |
| Usual care | 1.47 (0.34; 2.61) | 1.11 (-0.12; 2.34) | 0.94 (-0.83; 2.71) | 0.70 (-0.07; 1.47) | 0.26 (-0.31; 0.84) | 0.04 (-1.01; 1.08) | Usual care |

Table 5 League table of network meta-analysis for the effects of different mind-body exercises on left ventricular ejection fraction (LVEF)

| **Interventions** | **Yijinjing** | **Yoga** | **TaiChi** | **Baduanjin** | **Exercise** | **Usual care** | **Liuzijue** |
| --- | --- | --- | --- | --- | --- | --- | --- |
| Yijinjing | Yijinjing | . | . | . | . | 1.17 (0.57; 1.77) | . |
| Yoga | 0.61 (-0.24; 1.46) | Yoga | . | . | . | 0.56 (-0.04; 1.16) | . |
| Tai Chi | 0.94 (0.24; 1.64) | 0.33 (-0.37; 1.02) | Tai Chi | 0.29 (-0.33; 0.90) | -0.05 (-0.56; 0.46) | 0.23 (-0.16; 0.61) | . |
| Baduanjin | 0.95 (0.28; 1.61) | 0.33 (-0.33; 1.00) | 0.01 (-0.41; 0.42) | Baduanjin | . | 0.24 (-0.05; 0.54) | . |
| Exercise | 1.01 (0.20; 1.82) | 0.40 (-0.41; 1.21) | 0.07 (-0.39; 0.54) | 0.06 (-0.53; 0.66) | Exercise | -0.37 (-1.44; 0.69) | 0.36 (-0.65; 1.36) |
| Usual care | 1.17 (0.57; 1.77) | 0.56 (-0.04; 1.16) | 0.23 (-0.12; 0.59) | 0.23 (-0.06; 0.52) | 0.16 (-0.38; 0.71) | Usual care | 0.73 (-0.32; 1.79) |
| Liuzijue | 1.62 (0.51; 2.72) | 1.00 (-0.10; 2.11) | 0.68 (-0.26; 1.61) | 0.67 (-0.30; 1.63) | 0.60 (-0.31; 1.52) | 0.44 (-0.49; 1.37) | Liuzijue |

Table 6 League table of network meta-analysis for the effects of different mind-body exercises on NT-proBNP levels

| **Interventions** | **Yijinjing** | **Yoga** | **Exercise** | **Liuzijue** | **TaiChi** | **Baduanjin** | **Breathing** | **Usual care** |
| --- | --- | --- | --- | --- | --- | --- | --- | --- |
| Yijinjing | Yijinjing | . | . | . | . | . | . | -2.31 (-2.93; -1.70) |
| Yoga | -1.41 (-2.19; -0.64) | Yoga | -0.06 (-0.79; 0.67) | . | . | . | . | -1.18 (-1.73; -0.63) |
| Exercise | -1.98 (-2.72; -1.23) | -0.56 (-1.07; -0.05) | Exercise | 0.06 (-0.90; 1.01) | 0.14 (-0.31; 0.59) | . | . | -0.27 (-1.29; 0.75) |
| Liuzijue | -1.95 (-3.01; -0.89) | -0.54 (-1.48; 0.40) | 0.03 (-0.82; 0.88) | Liuzijue | . | . | . | -0.33 (-1.32; 0.66) |
| Tai Chi | -2.01 (-2.68; -1.34) | -0.60 (-1.09; -0.10) | -0.03 (-0.42; 0.35) | -0.06 (-0.93; 0.81) | Tai Chi | -0.09 (-0.62; 0.45) | . | -0.22 (-0.49; 0.05) |
| Baduanjin | -2.02 (-2.68; -1.36) | -0.61 (-1.12; -0.09) | -0.04 (-0.51; 0.42) | -0.07 (-0.95; 0.81) | -0.01 (-0.33; 0.31) | Baduanjin | . | -0.29 (-0.52; -0.06) |
| Breathing | -2.24 (-3.09; -1.39) | -0.83 (-1.57; -0.08) | -0.27 (-0.98; 0.45) | -0.29 (-1.33; 0.74) | -0.23 (-0.87; 0.40) | -0.22 (-0.85; 0.40) | Breathing | -0.07 (-0.66; 0.51) |
| Usual care | -2.31 (-2.93; -1.70) | -0.90 (-1.36; -0.44) | -0.34 (-0.75; 0.07) | -0.36 (-1.22; 0.49) | -0.30 (-0.55; -0.06) | -0.30 (-0.52; -0.07) | -0.07 (-0.66; 0.51) | Usual care |

Table 7 Summary of SUCRA values and efficacy rankings for different intervention protocols across all clinical outcomes

| **Interventions** | **MLHFQ** | **6MWD** | **Peak VO2​** | **NT-proBNP** | **LVEF** |
| --- | --- | --- | --- | --- | --- |
| Yijinjing | 0.978 (Rank 1) | 0.660 (Rank 2) | 0.858 (Rank 1) | 1.000 (Rank 1) | 0.98... (Rank 1) |
| Meditation | 0.536 (Rank 5) | 0.675 (Rank 1) | NA | NA | NA |
| Liuzijue | 0.900 (Rank 2) | 0.600 (Rank 4) | NA | 0.468 (Rank 5) | 0.08... (Rank 10) |
| Yoga | 0.597 (Rank 4) | 0.476 (Rank 6) | 0.728 (Rank 2) | 0.831 (Rank 2) | 0.75... (Rank 2) |
| Baduanjin | 0.293 (Rank 8) | 0.640 (Rank 3) | 0.573 (Rank 4) | 0.443 (Rank 7) | 0.51... (Rank 4) |
| Tai Chi | 0.662 (Rank 3) | 0.500 (Rank 5) | 0.343 (Rank 6) | 0.452 (Rank 6) | 0.52... (Rank 3) |
| Pilates | NA | NA | 0.646 (Rank 3) | NA | NA |
| Exercise | 0.398 (Rank 6) | 0.357 (Rank 8) | 0.205 (Rank 7) | 0.479 (Rank 4) | 0.43... (Rank 6) |
| Usual care | 0.069 (Rank 10) | 0.196 (Rank 9) | 0.148 (Rank 8) | 0.096 (Rank 8) | 0.21... (Rank 8) |


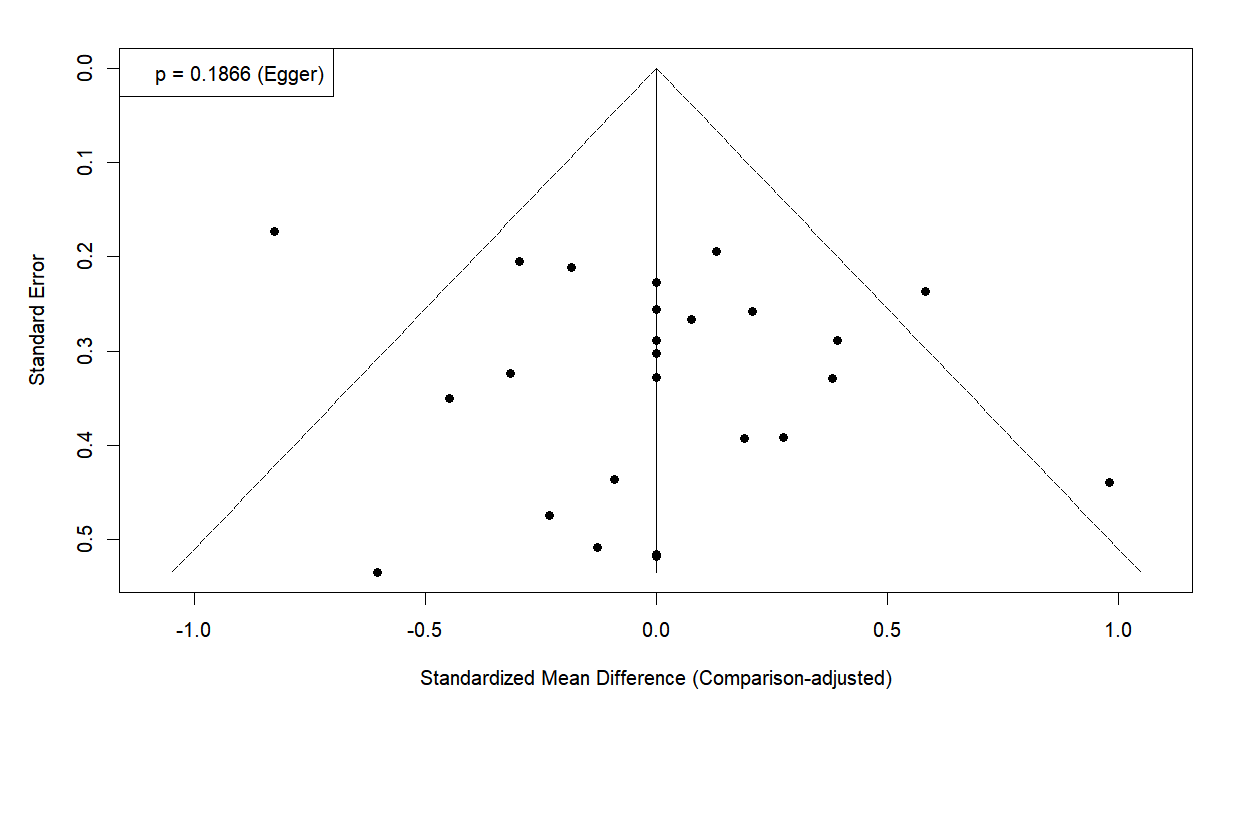


Appendix Figure 1 Comparison-adjusted funnel plot and Egger's test results for publication bias in the network meta-analysis of quality of life (MLHFQ)


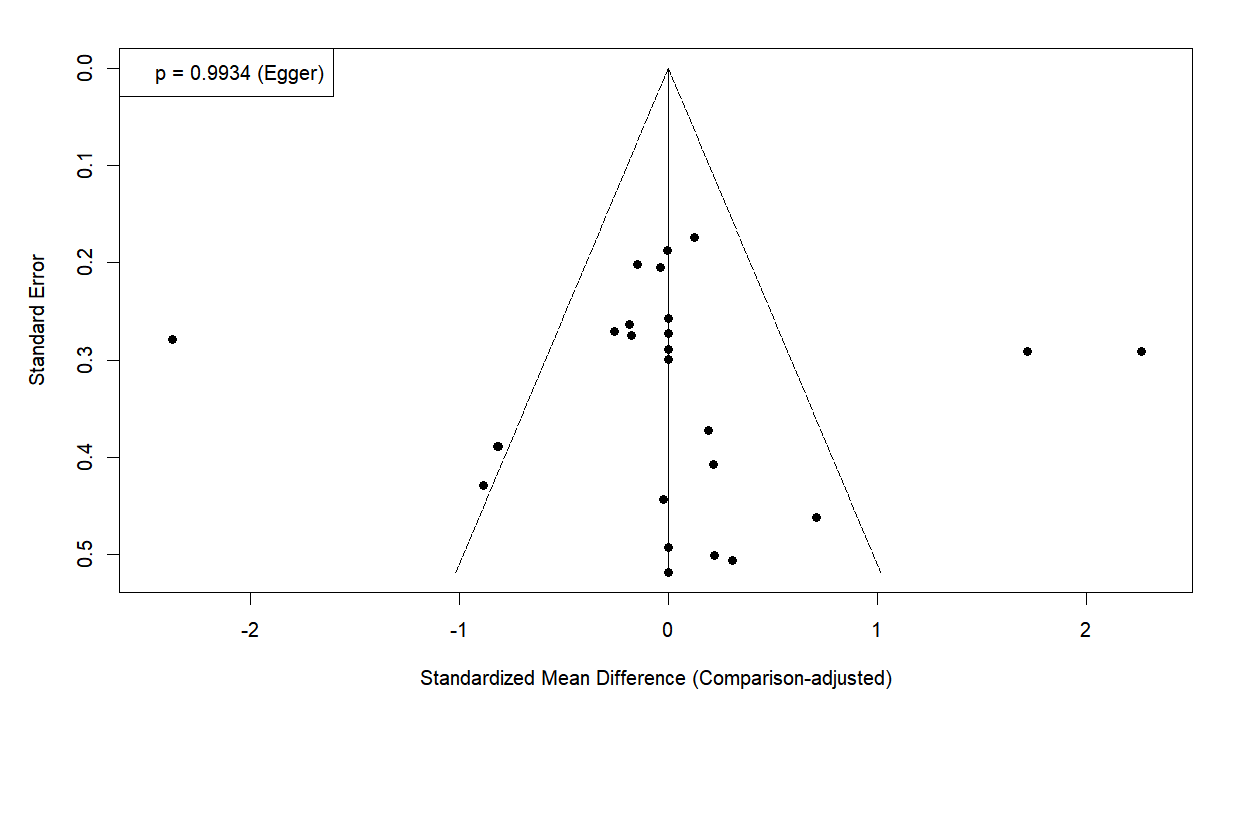


Appendix Figure 2 Comparison-adjusted funnel plot and Egger's test results for publication bias in the network meta-analysis of exercise tolerance (6MWD)


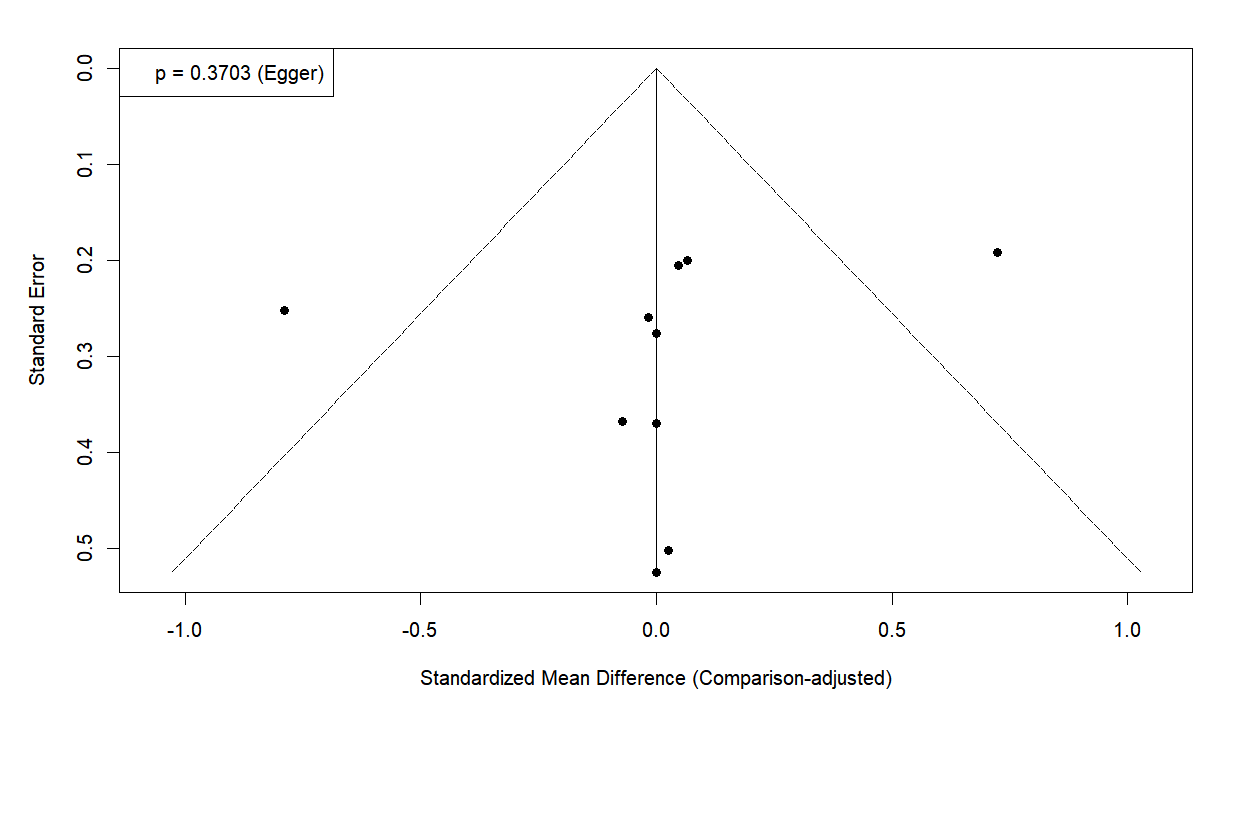


Appendix Figure 3 Comparison-adjusted funnel plot and Egger's test results for publication bias in the network meta-analysis of cardiorespiratory fitness (Peak VO_2_)


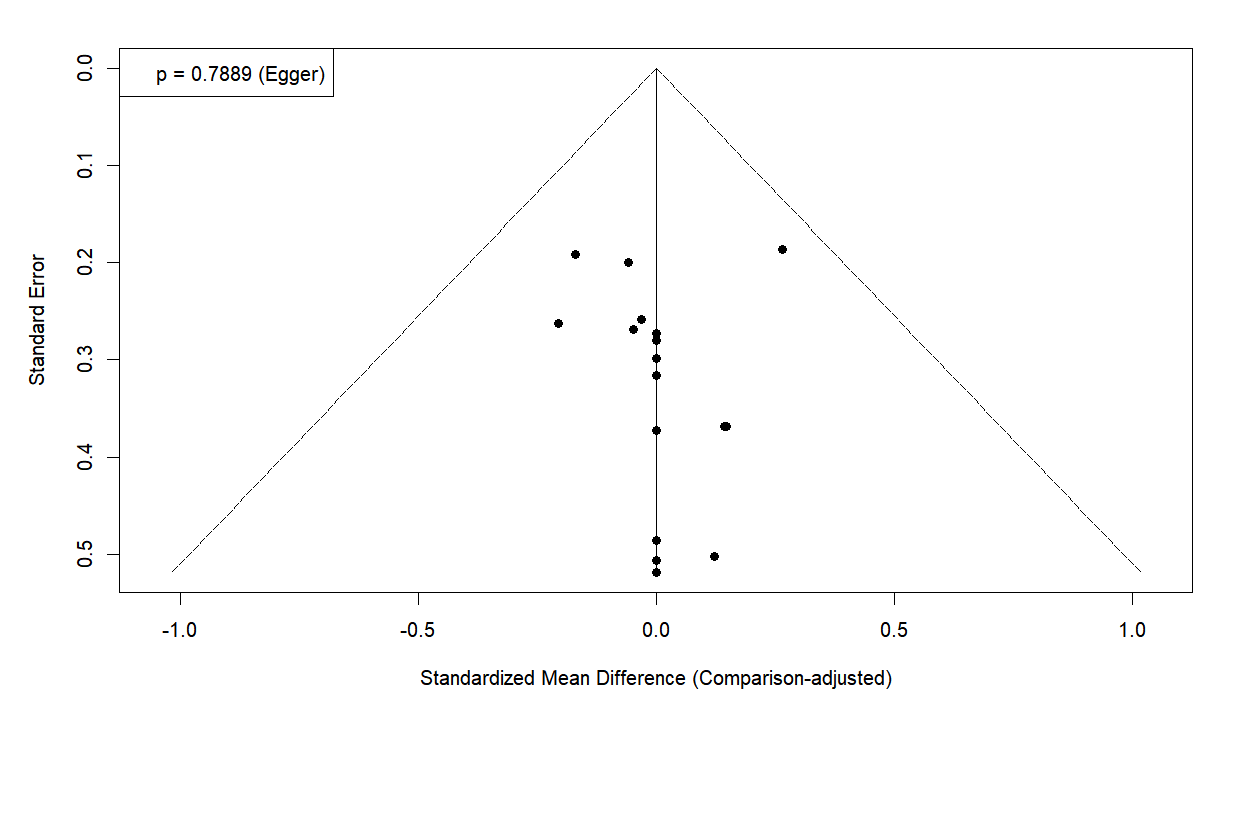


Appendix Figure 4 Comparison-adjusted funnel plot and Egger's test results for publication bias in the network meta-analysis of NT-proBNP levels


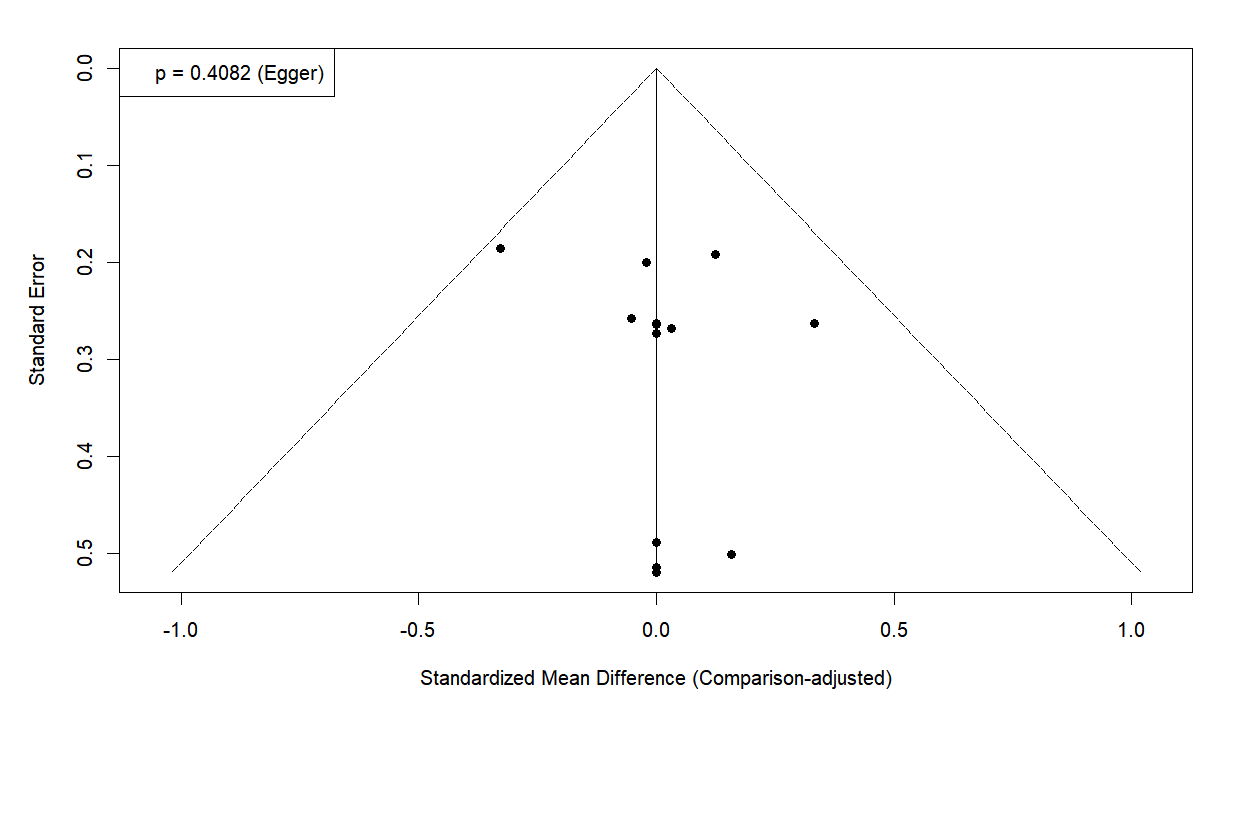


Appendix Figure 5 Comparison-adjusted funnel plot and Egger's test results for publication bias in the network meta-analysis of left ventricular ejection fraction (LVEF)


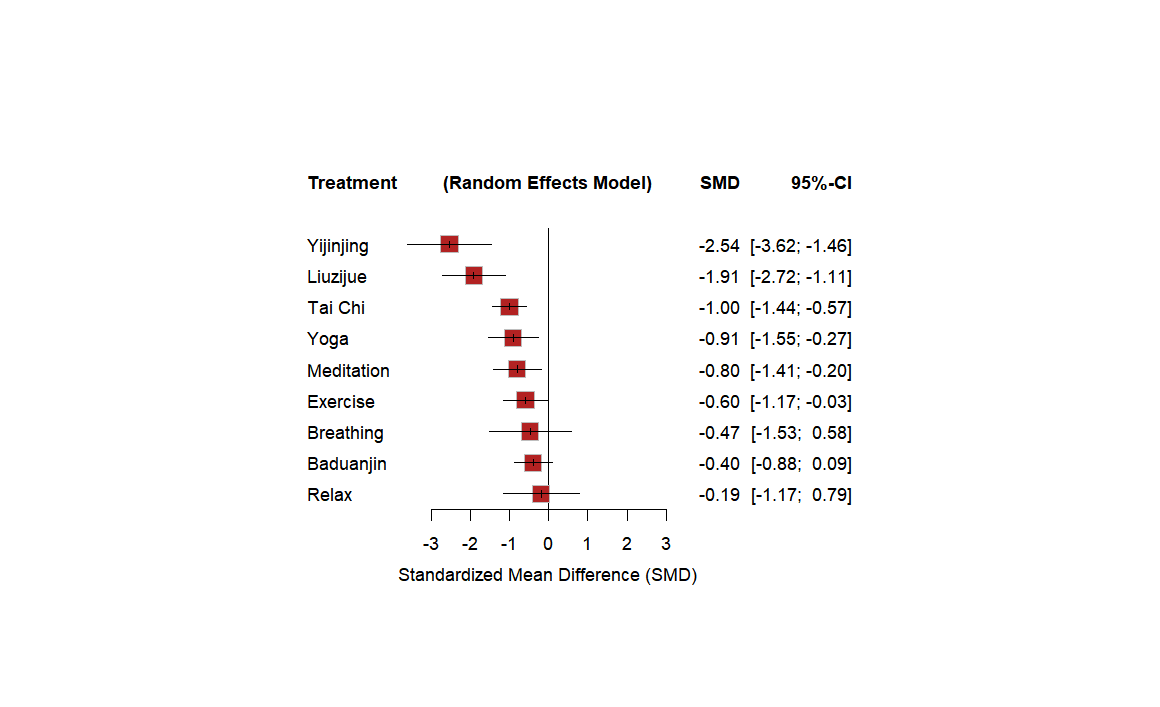


Appendix Figure 6 Forest plot of the effects of mind-body exercise interventions versus usual care (UC) on quality of life (MLHFQ)


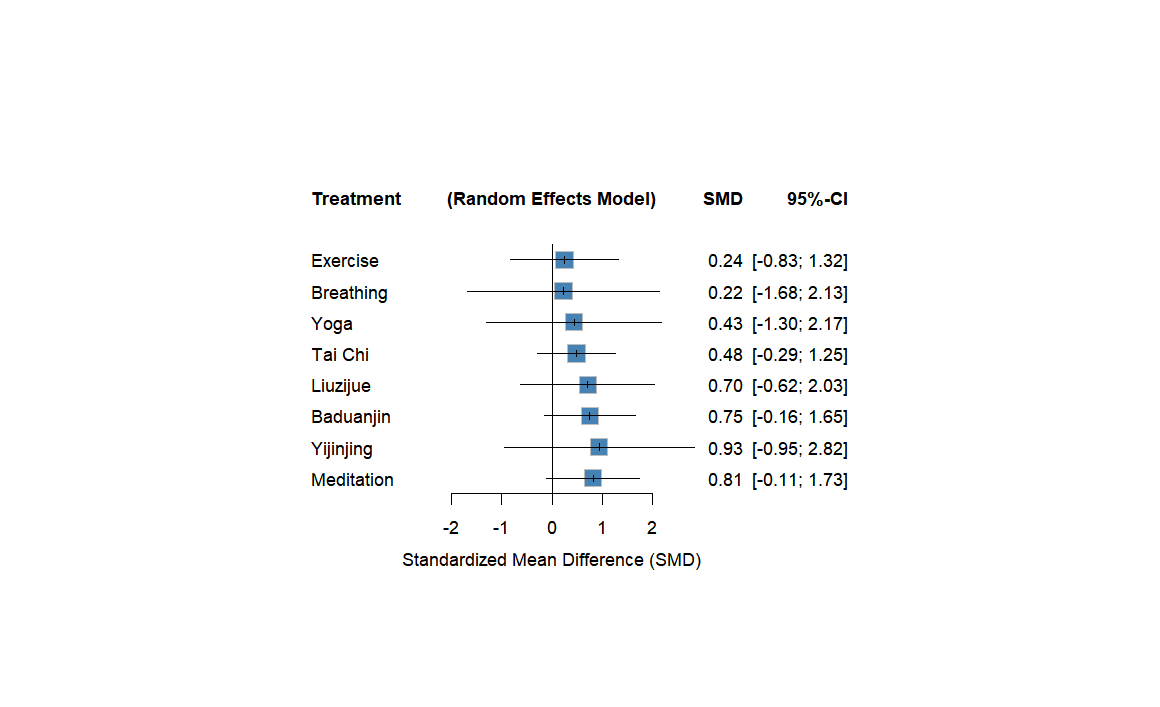


### Appendix Figure 7 Forest plot of the effects of mind-body exercise interventions versus usual care (UC) on exercise tolerance (6MWD)


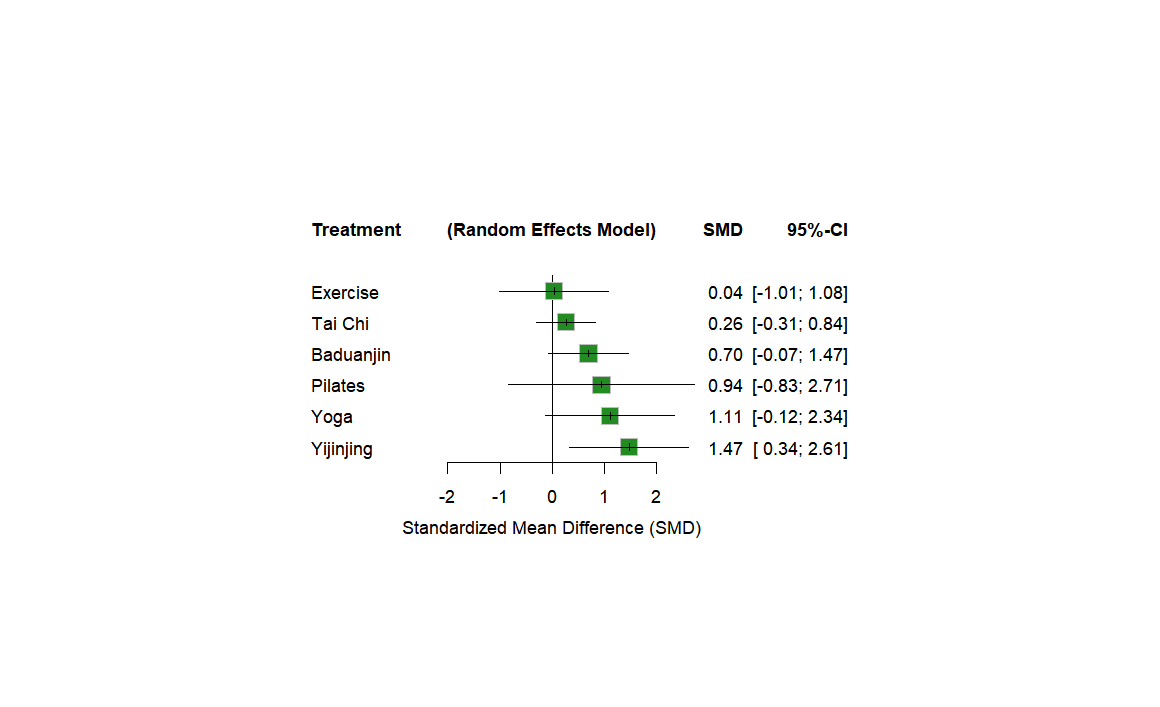


### Appendix Figure 8 Forest plot of the effects of mind-body exercise interventions versus usual care (UC) on cardiorespiratory fitness (Peak VO2)


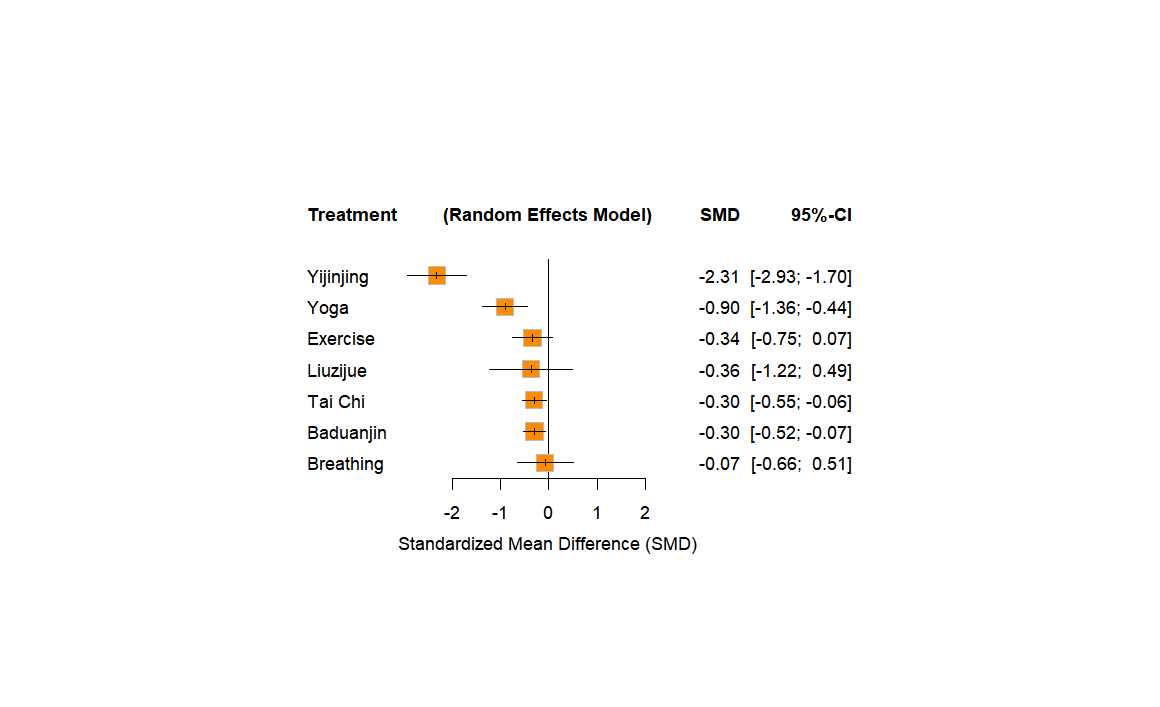


Appendix Figure 9 Forest plot of the effects of mind-body exercise interventions versus usual care (UC) on NT-proBNP levels
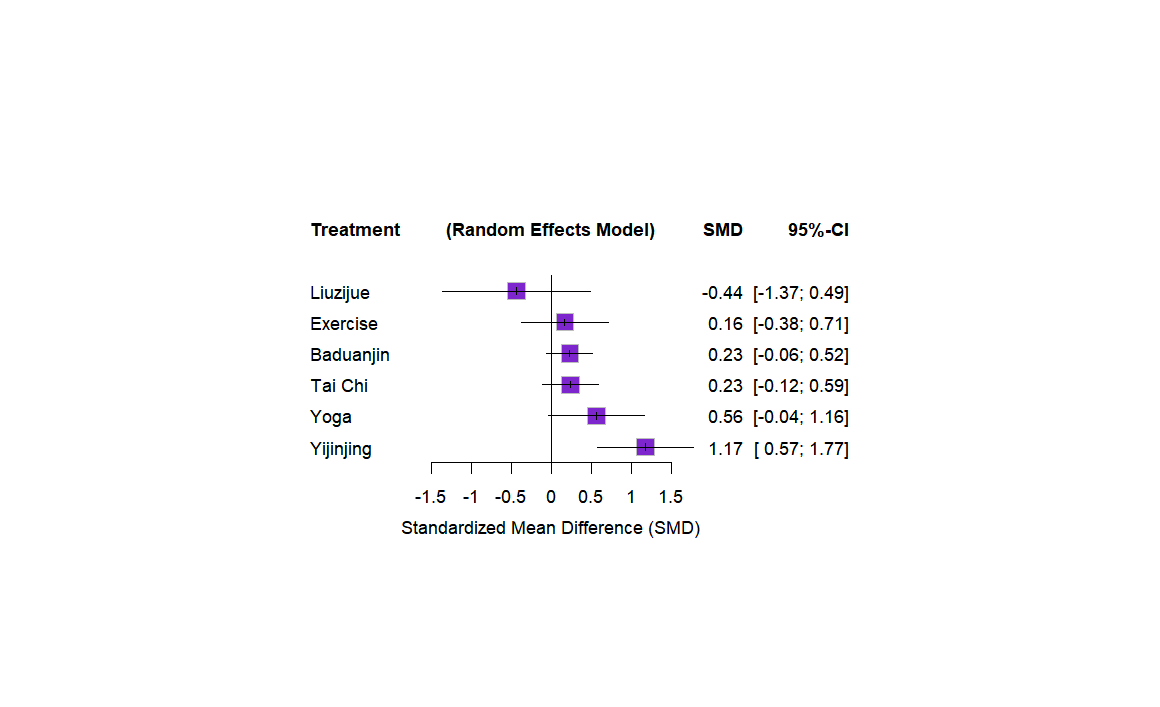


Appendix Figure 10 Forest plot of the effects of mind-body exercise interventions versus usual care (UC) on left ventricular ejection fraction (LVEF)


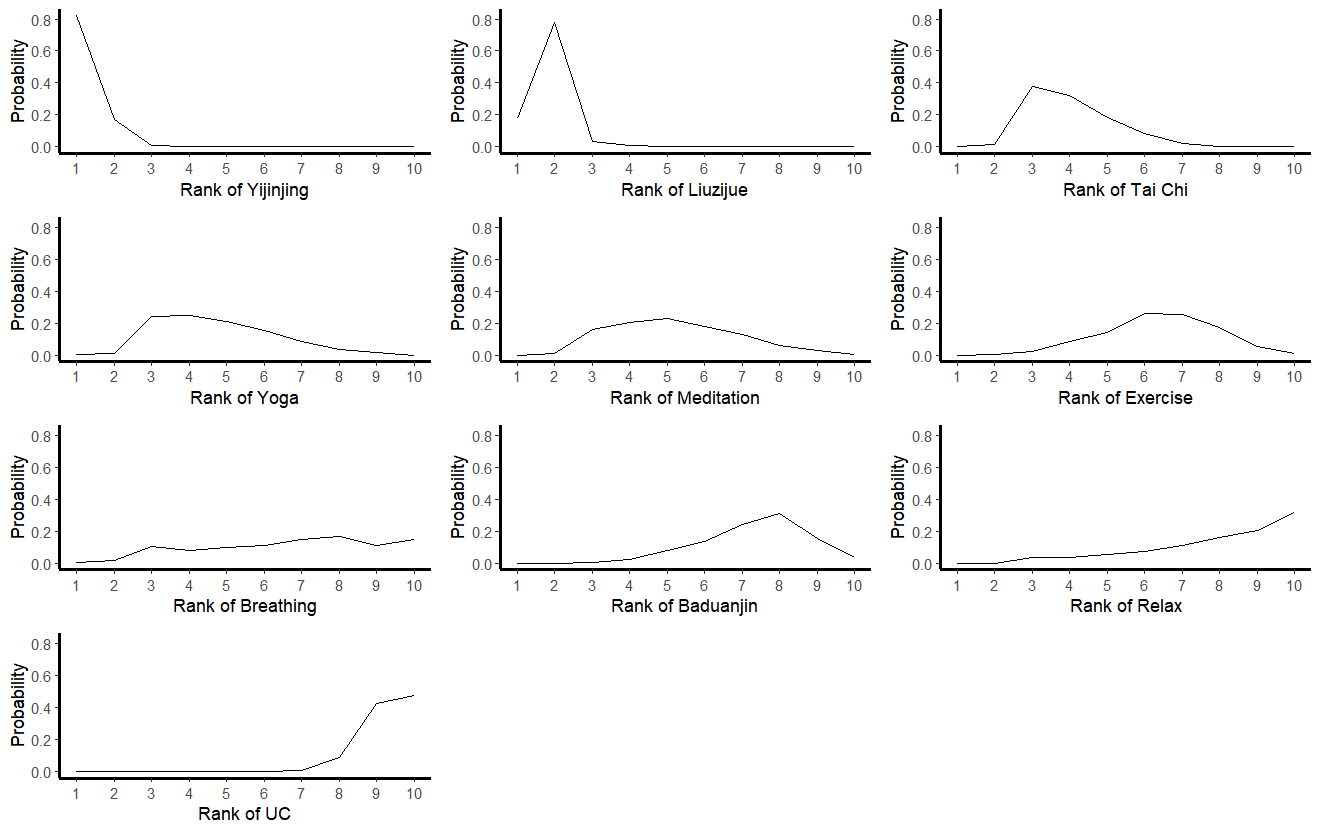


Appendix Figure 11 Surface under the cumulative ranking curve (SUCRA) plots for different intervention protocols on quality of life (MLHFQ)
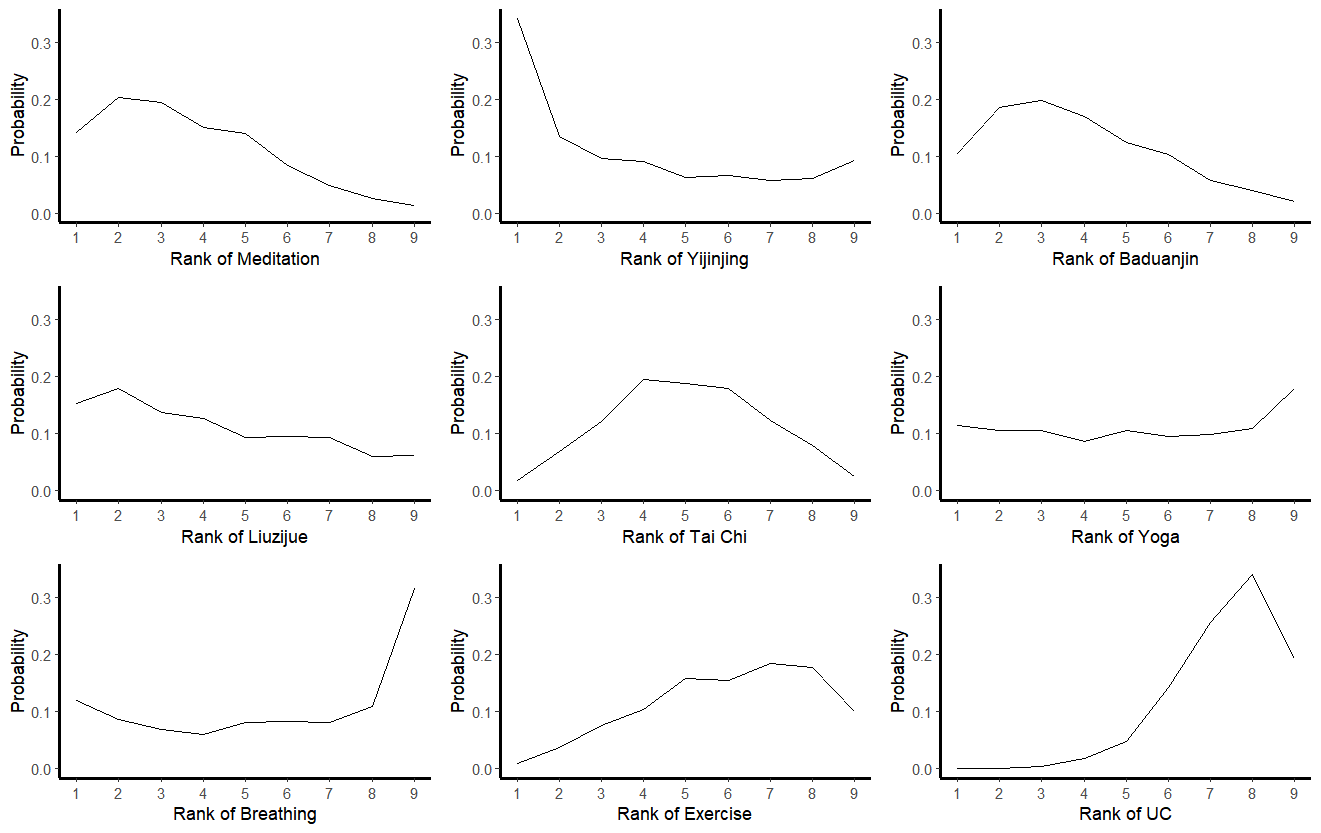


Appendix Figure 12 Surface under the cumulative ranking curve (SUCRA) plots for different intervention protocols on exercise tolerance (6MWD)


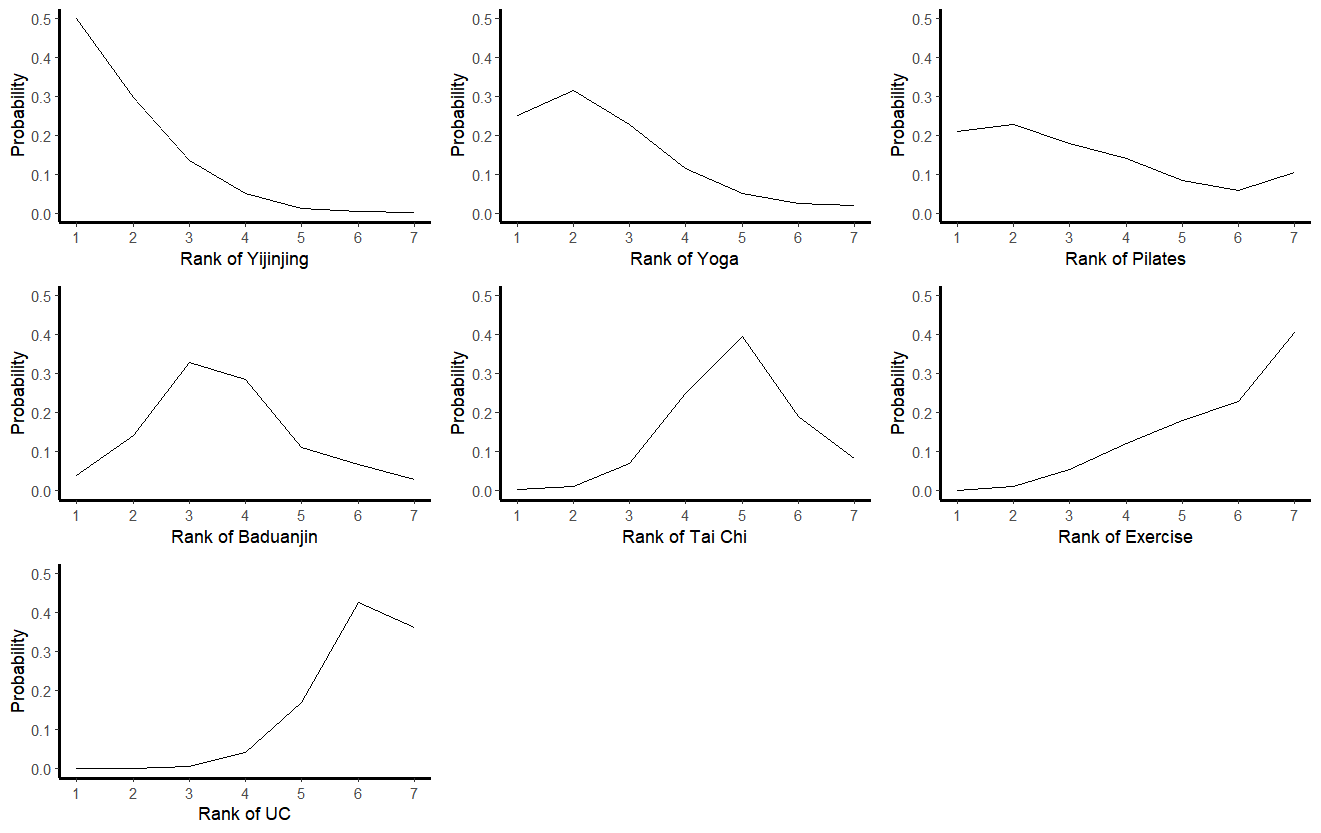


Appendix Figure 13 Surface under the cumulative ranking curve (SUCRA) plots for different intervention protocols on cardiorespiratory fitness (Peak VO_2_)
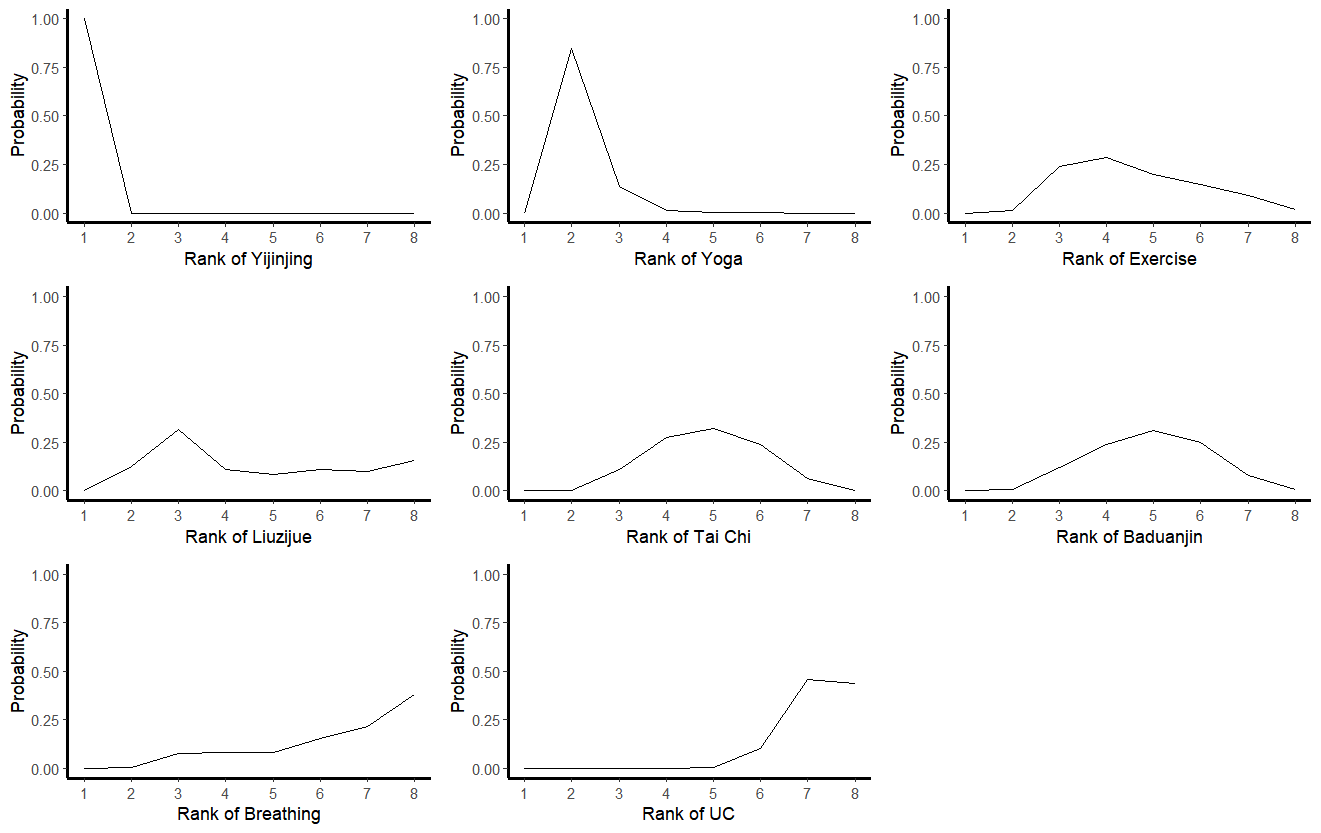


Appendix Figure 14 Surface under the cumulative ranking curve (SUCRA) plots for different intervention protocols on NT-proBNP levels


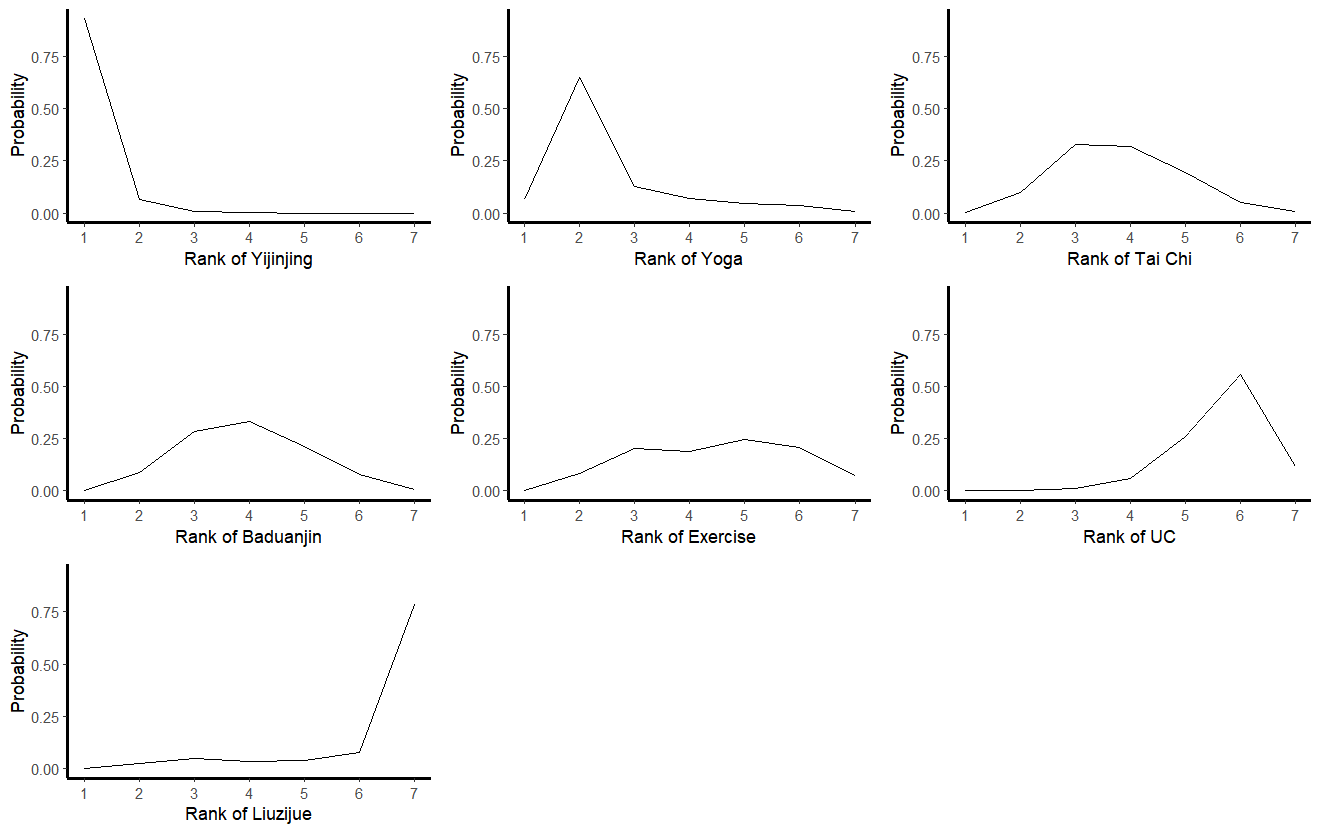


Appendix Figure 15 Surface under the cumulative ranking curve (SUCRA) plots for different intervention protocols on left ventricular ejection fraction (LVEF)
